# Deep longitudinal multiomics profiling reveals two biological seasonal patterns in California

**DOI:** 10.1038/s41467-020-18758-1

**Published:** 2020-10-01

**Authors:** M. Reza Sailani, Ahmed A. Metwally, Wenyu Zhou, Sophia Miryam Schüssler-Fiorenza Rose, Sara Ahadi, Kevin Contrepois, Tejaswini Mishra, Martin Jinye Zhang, Łukasz Kidziński, Theodore J. Chu, Michael P. Snyder

**Affiliations:** 1 Department of Genetics, Stanford University, Stanford, CA 94305 USA; 2 Department of Electrical Engineering, Stanford University, Stanford, CA 94305 USA; 3 Department of Bioengineering, Stanford University, Stanford, CA 94305 USA; 4 Department of Pediatrics, Division of Allergy and Immunology, Stanford University, Stanford, CA 94305 USA

**Keywords:** Biological techniques, Ecology, Immunology, Microbiology, Molecular biology

## Abstract

The influence of seasons on biological processes is poorly understood. In order to identify biological seasonal patterns based on diverse molecular data, rather than calendar dates, we performed a deep longitudinal multiomics profiling of 105 individuals over 4 years. Here, we report more than 1000 seasonal variations in omics analytes and clinical measures. The different molecules group into two major seasonal patterns which correlate with peaks in late spring and late fall/early winter in California. The two patterns are enriched for molecules involved in human biological processes such as inflammation, immunity, cardiovascular health, as well as neurological and psychiatric conditions. Lastly, we identify molecules and microbes that demonstrate different seasonal patterns in insulin sensitive and insulin resistant individuals. The results of our study have important implications in healthcare and highlight the value of considering seasonality when assessing population wide health risk and management.

## Abstract (web summary)

Seasonal patterns of molecular markers in humans have not been extensively studied. Here, the authors combine host components (transcriptome, metabolome, proteome, immunome, clinical lab tests) and microbiome to profile 105 individuals, identifying over 1000 markers in two major seasonal patterns.

## Introduction

The environment is a key factor in human health, and seasonal changes in particular have been associated with human conditions and diseases. For example, mortality rates in the U.S. show a notable seasonality with rates in winter 25% higher than in summer^1^. Other human phenotypes associated with seasons are allergies, autoimmune conditions^2^, and cardiovascular diseases^3^ as well as psychiatric disorders^4,5^. In addition, a series of large-scale population-based studies revealed that systolic and diastolic blood pressures were higher in winter than in summer^6–8^.

Photoperiodism has been considered to be one of major cues for organismal responses to seasons^9^. It enables plants and animals to measure environmental day length to ascertain time of year^10^. For example, flowering time in response to photoperiod and temperatures has been well studied using model plants (Arabidopsis and rice)^9,11^. The underlying mechanism for photoperiod perception is measured daily through interactions between the internal circadian clock and the external light–dark cycle^9,11,12^.

Although external cues and diseases have been monitored, little is known about how human biological and physiological processes change in response to the seasons. The effect of seasonal variation on humans has been primarily focused on gene expression data^13–15^. These studies are limited in the number of individuals and the time span of the study, the intensity of sample collection across the year and the depth of experiments and features that are quantified. Moreover, they do not assess whether seasonal changes might affect individuals with different disease conditions, for example, insulin sensitive (IR) versus insulin resistant (IR) individuals (insulin resistance is often associated with Type 2 diabetes). In addition, most studies choose to study seasonal changes based on the current paradigm of four equal-sized seasons, which is an arbitrary window and may not reflect natural biological patterns in many parts of the country. Understanding human biological patterns regardless of arbitrary calendar dates is expected to be important to improve disease risk prediction, susceptibility, and diagnostics.

We and others have demonstrated the value of deep longitudinal profiling to decipher complex physiological processes in humans including diabetes onset, viral infection and detoxification pattern^16–18^.

In this work, we leverage the power of multiomics profiling to examine the calendar patterns of biomolecular features in a systematic fashion to elucidate (1) the seasonal patterns of diverse individual molecules and pathways, (2) the overall patterns of biological changes (i.e., how many distinct major patterns exist and when do they occur independently of the arbitrary four seasons), and (3) molecular and microbial differences between insulin sensitive versus insulin resistant individuals throughout the year. By performing deep sampling and omics profiling for 105 individuals for up to 4 years, we discover more than 1000 molecular seasonal variation changes in both microbiome and host molecules; these changes group into two major patterns. We further demonstrate differential seasonal fluctuations depending on insulin resistance status. These findings have important implications for human health.

## Results

### Cohort and data description

**Fig. 1:**
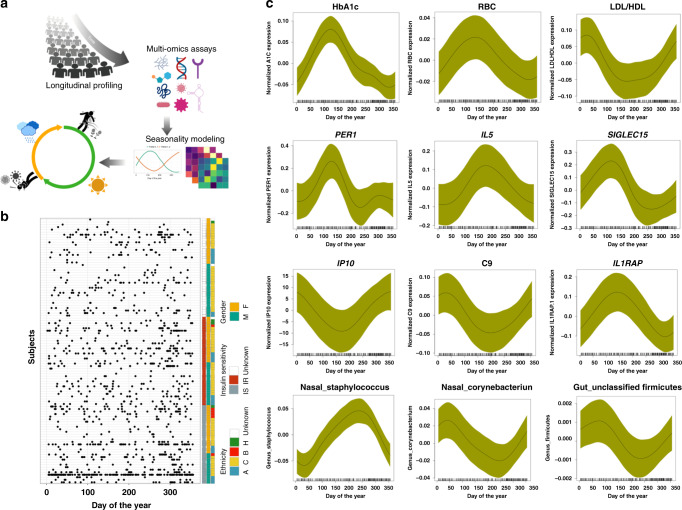
Study design and seasonality. **a**. Integrative personal omics profiling (iPOP) cohort sampling and data collection for seasonal analyses. Omic assays included immune molecules profiling using Luminex assay, proteomics using sequential windowed acquisition of all theoretical fragment ion mass spectrometry (SWATH-MS), metabolomics using liquid chromatography (LC)—mass spectrometry, transcriptomics and microbial profiling (gut and nasal) using next-generation sequencing, in conjunction with clinical lab tests and meteorological measurements. **b** Subjects and sampling timepoints for each individual, as well as ethnicity (A: Asian, B; Black, C; Caucasian), insulin sensitive (IS) and insulin resistance (IR), and gender information (M: Male, F: Female). **c** Examples of omics analytes with seasonal patterns (transcripts, cytokines, metabolites, proteins, clinical lab tests, gut and nasal microbiome). The *X-*axis shows the days of the year (1–365 days) and *Y-*axis shows the normalized expression/abundance values. The samples are collected up to 4 years and aggregated and mapped to 1-year-long time frame. The shaded area represents 95% confidence bounds computed as ±1.96 standard deviation of model coefficients. Standard deviations were derived from a maximum likelihood fit.

In order to examine seasonal changes of human molecular data, we leveraged the power of longitudinal multiomics data from profiling of 105 individuals (55 women and 50 men) with ages ranging from 25 to 75 years old (Fig. 1a; Supplementary Table 1). This cohort was generally healthy and well characterized for glucose dysregulation using annual oral glucose tolerance tests (OGTTs), insulin resistance measuring steady-state plasma glucose (SSPG), fasting glucose and hemoglobin A1c (HbA1c; an indicator of the average level of blood glucose over the past 100 days)^19^ as well as quarterly sample collections with measurements of transcriptomes (from peripheral blood mononuclear cells), proteome and metabolome from plasma, targeted cytokine and growth factor assays using serum. Nasal and gut microbiomes were analyzed using 16S rRNA sequencing providing information at the genus level and host exome sequencing was performed once from PBMCs (Fig. 1b). Moreover, 51 clinical laboratory tests were acquired on each visit and they were aligned to the meteorological data (e.g. air temperature), pollen counts (e.g. mold spores, grass pollens, tree pollens, weed pollens) and airborne fungi from the San Francisco bay area. In total, there were 902 visits (average across different types of omes‘) from which samples were drawn over up to 4 years (see “Methods”). The sample collections were generally evenly distributed throughout the year (Fig. 1b). Nearly all individuals lived in the San Francisco Bay Area with the exception of three individuals who lived in Southern California and frequented the Bay area (Supplementary Data 1). Participants in our study were well characterized for steady-state plasma glucose (SSPG) using the modified insulin suppression test^20^, in which 31 participants were insulin sensitive (SSPG < 150 mg/dL), and 35 were insulin resistant (SSPG ≥ 150 mg/dL) (Supplementary Table 1).

### Seasonal changes of diverse biological molecules

**Fig. 2:**
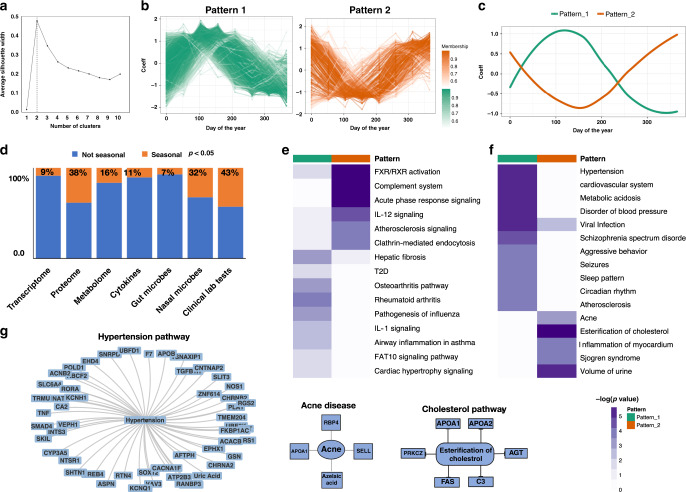
Multiomics seasonal clusters and biological pathways. **a** Silhouette width plot for identifying the optimal number of clusters for all the host omics analytes combined as well as individual omes‘ (Elbow plots in Supplementary Fig. 2). The *X*-axis shows the number of clusters (k) and the *Y*-axis shows average silhouette width. The silhouette plot shows the silhouette coefficient over values of *k* clusters ranging from 1 to 10. This plot shows the highest average silhouette coefficient occurring at *k* = 2. **b** Fuzzy C-means clustering of seasonal patterns for all omics analytes combined. The *X*-axis shows days of the year (1-365 days) and the *Y*-axis shows normalized *GAMM* coefficients (omics pattern one is shown in green color and omics patterns two is shown in orange color). **c** Summarized patterns for all the omics analytes combined as well as individual omes‘ (Supplementary Fig. 3) (omics pattern one is shown in green color and omics pattern two is shown in orange color). **d** Summary of proportion of omics analysts with seasonality effects (*GAMM* model likelihood ratio test *p*-value ≤ 0.05) per ome. **e** Integrative canonical pathway analysis and **f** Integrative disease analysis of omics analytes and their enrichment with seasonal patterns 1 and 2. The heatmap plot shows the −log_10_ (adj-*p* values). **g** Examples of disease enrichment analysis of seasonal patterns. Hypertension, acne and esterification of cholesterol pathways are shown.

We first systematically searched for molecules that fluctuate throughout the year. We applied a generalized additive mixed model (GAMM) (see “Methods”) in order to detect molecules with seasonality effects (*GAMM* model likelihood ratio test *p*-value ≤ 0.05). We identified seasonal patterns for 898 transcripts, 119 metabolites, 116 proteins, 22 clinical lab tests, seven cytokines, seven gut microbial taxa, and 23 nasal microbial taxa (*GAMM* model likelihood ratio test *p*-value ≤ 0.05; Fig. 2d, Supplementary Table 1, Supplementary Data 2). Our computational analysis detected expected molecules known to exhibit seasonal patterns such as HbA1c^19^ (*p*-value = 1.27E − 08), which peaks in spring and summer, and is low in winter^19^. We also found that RBC^21^ (red blood cells) (*p*-value = 0.029) and RDW (Red Blood Cell Distribution Width)^22^ (*p*-value = 0.002) follow a similar seasonal pattern. HDL (High-density lipoprotein)^23^ (*p*-value = 0.025) peaks in summer, and the LDL/HDL ratio (*p*-value = 0.003) peaks in winter (Fig. 1c). Also, *PER1* (period circadian regulator 1), the primary circadian pacemaker in the mammalian brain^24^, shows seasonal effects (*p*-value=0.003) with highest expression level in spring. *PER1* belongs to a family of genes responsible for the circadian rhythms of locomotor activity, metabolism, and tightly involved in photo- and thermo-periodic measurements^25,26^.

Our analysis also revealed a number of novel molecules with seasonality variations. C2, C9, IL5, *SIGLEC15*, and *IL1RAP* are examples of molecules with roles in immunity, inflammation and allergy that demonstrate seasonal effects (Fig. 1c; Supplementary Data 2). Although the majority of omics molecules peaked once during the year, we found multiple molecules that peaked twice or thrice such as *CTTNBP2*, *COQ10A*, and gut microbial genus Holdemania (Supplementary Fig. 1). Thus, a large number of molecules undergo seasonal changes with a variety of different patterns.

### Two predominant seasonal patterns in California

Presently we think of seasons as four equally partitioned periods arbitrarily set by the calendar. To determine if general seasonal patterns of molecules could be observed and how many classes might exist in our California cohort, we performed fuzzy C-means clustering with Silhouette criterion on both normalized multiomics data as well as individual omes’ to determine the number of clusters (see “Methods”; Fig. 2a; Supplementary Fig. 2). Rather than four seasonal patterns, two distinct clusters of omics molecules both at the level of all omes’ combined as well as each single ome were observed (Fig. 2a–c; Supplementary Fig. 3; Supplementary Data 3). Interestingly, omics seasonal pattern one peaks in late April, whereas omics seasonal pattern two peaks in December and drops in March through July. Pattern one corresponds to late spring, a period of high pollen count and end of the California rainy season, and pattern two peaks in late fall and early winter, a period of high viral infection incidents. Individual omes‘ exhibited similar but slightly shifted patterns, depending upon the ome (Fig. 2c).

### Integrative pathway analysis of seasonal signatures

To obtain a better understanding of the biological processes and human diseases associated with each seasonal pattern, we performed pathway and disease enrichment analyses using Ingenuity pathway analysis (see “Methods”) using all the 1133 omics molecules that showed significant seasonal effects (*p* ≤ 0.05) (Supplementary Data 2).

Pathways and their associated diseases that change during the two omics seasonal patterns are shown in Fig. 2 (Fig. 2e, f; Supplementary Data 4; Supplementary Table 2; adjusted *p* ≤ 0.05). Interestingly, we found disorders related to blood pressure, hypertension, and cardiovascular disease to be associated with seasonal omics pattern one where the omics molecules have the highest expression level in spring/summer. Other notable pathways and diseases associated with pattern one are schizophrenia spectrum disorder, sleep pattern, and seizure (Fig. 2e). We also discovered that transcripts from 12 collagen genes show a strong match with pattern one (Supplementary Fig. 4). Collagen plays a structural role by contributing to the molecular architecture, shape, and mechanical properties of tissues, such as the tensile strength in skin and the resistance to traction in ligaments^27^.

**Fig. 3:**
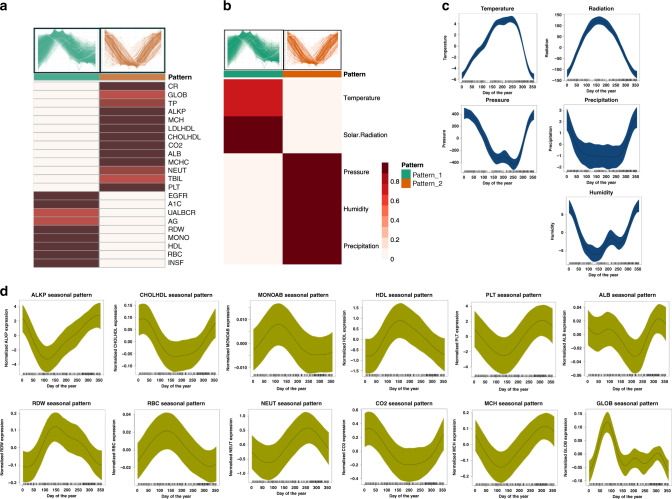
Clinical lab tests and meteorological data correlate with omics seasonal patterns. Correlation of (**a**) clinical lab tests and (**b**) meteorological measurements with the two omics seasonal patterns are shown. The heatmap shows cluster membership values (which are on a scale of 0 and 1) based on existing cluster centroids. Seasonal patterns of (**c**) meteorological measurements and (**d**) clinical lab tests are shown (omics pattern one is shown in green color and omics pattern two is shown in orange color). The *X*-axis shows the days of the year (1–365 days) and *Y*-axis show the normalized measurement values. The shaded area represents 95% confidence bounds computed as ±1.96 standard deviation of model coefficients. Standard deviations were derived from a maximum likelihood fit.

Pattern two is associated with acute phase response, clathrin-mediated endocytosis, esterification of cholesterol, volume of urine and acne, as well as other pathways (Fig. 3a, b). The acute phase response (Supplementary Fig. 5) is upregulated over fall and winter and is a rapid inflammatory response that provides protection against microorganisms (bacteria, viruses, etc.). It involves an increase in pro-inflammatory cytokines (IP10, IL1, IL1R1, IL1RAP, IL6) and a change in concentration of plasma acute phase proteins and complement system (C2, C3, C9). Clathrin-mediated endocytosis pathway (Fig. 2e, f) is also associated with pathogen-influenced signaling and is a major gateway for the internalization of nutrients, hormones and other signaling molecules from the plasma membrane into intracellular compartments. The complement system (Fig. 2e, f; Supplementary Fig. 6) is a cascade of enzyme activations that bridges innate and acquired immune systems and is involved in clearance of immune complexes, activation of inflammation and augmenting the antibody response^28,29^. Complement system defects have been found in autoimmune disorders such as systemic lupus erythematosus that can affect the joints, skin, kidneys, blood and lungs. These results indicate that biological processes and their associated diseases correlate with the two major seasonal patterns.

### Correlation of seasonal patterns with clinical lab tests and meteorological measurements

We next conducted the seasonality analysis for 51 clinical laboratory measures (Supplementary Data 6) and identified health markers with significant seasonality components (Figs. 1c; 3a, d; Supplementary Data 2; *p* ≤ 0.05). We further correlated these markers with the two omics seasonal patterns described above (Fig. 3a). As mentioned above, we observed that A1c (Hemoglobin A1c), as well as insulin, RDW (red blood cell distribution width), RBC (red blood cell counts), MONO (monocytes), UALB (urine albumin), and EGFR (estimated glomerular filtration rate) correlated with omics seasonal pattern one, whereas LDLHDL (LDL/HDL ratio), CHOLHDL (cholesterol/HDL ratio), MCH (mean corpuscular hemoglobin), ALB (albumin), PLT (platelet count), ALKP (alkaline phosphatase), CR (creatinine), CO_2_ (carbon dioxide), GLOB (Globulin) and NEUTAB (neutrophil absolute count), AG (albumin/globulin ratio), TBIL (Total Bilirubin) and TP (total protein) correlated with omics seasonal pattern two (Fig. 3a, d). These findings highlight the extensive seasonality variation in clinical health biomarkers and the importance of considering seasonality components in interpreting health biomarkers in the clinic.

Since most of the individuals that participated in this study are residents of the San Francisco Bay Area, we further correlated omics seasonal patterns with meteorological measurements collected from this area (see “Methods”, Fig. 3b). Average air temperature and average solar radiation correlated with omics seasonal pattern one, whereas average air pressure, air humidity, and precipitation correlated better with pattern two (Fig. 3b).

### Seasonal gut and nasal microbial shifts

**Fig. 4:**
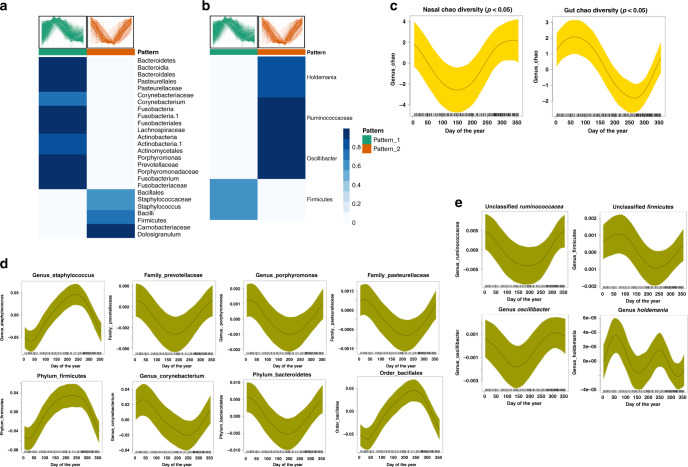
Nasal and gut microbial taxa correlate with omics seasonal patterns. Correlation of (**a**) nasal and (**b**) gut microbial taxa with the two omics seasonal patterns. The heatmap shows cluster membership values (which are on a scale of 0 and 1) based on existing cluster centroids (omics pattern one is shown in green color and omics pattern two is shown in orange color). **c** Gut and nasal microbiome diversity scores (by Chao diversity) over the year. The *Y*-axis shows the days of the year and *X*-axis shows normalized Chao diversity scores. The shaded area represents 95% confidence bounds computed as ±1.96 standard deviation of model coefficients. Standard deviations were derived from a maximum likelihood fit. **d** Gut microbial taxa and (**e**) nasal microbial taxa with seasonal patterns.

In order to detect microbial shifts over the two omics related patterns, we calculated GAMM seasonal coefficients for gut and nasal microbiome from the same individuals. We first measured the diversity of the gut and nasal microbiome by estimating Chao diversity index^30^. We discovered that the overall microbial diversity increased in winter compared to summer (*p* ≤ 0.05) in both nasal and gut microbiomes at all tested taxonomic levels (genus, family, order, class, phylum) (Fig. 4c). The correlation of gut and nasal microbial taxa with patterns one and two are shown in Fig. 4a, b. Overall, we observed more seasonal changes in nasal microbial taxa (23 microbial taxa) compared to the gut (four microbial taxa) (Supplementary Fig. 7a, b; Supplementary Data 2). Gut microbial taxa *Holdemania* genus, *Ruminococcaceae* genus*, Oscillibacter* genus as well as *Firmicutes* phylum show significant (*p* ≤ 0.05) seasonal components. These microbial taxa correlated with the omics seasonal pattern one (Fig. 4b). Of notable nasal bacterial taxa, we observed *Staphylococcus* genus*, Porphyromonas* genus, *Dolosigranulum* genus, *Corynebacterium* genus*, Bacteroidia* class*, Bacilli* class*, Actinobacteria* class, *and Pasteurellaceae* family show seasonal effects and correlate with either omics pattern one or two, depending upon the taxa (Fig. 4). Thus, the human nasal and gut microbiome undergoes extensive seasonal changes.

### Correlation of seasonal patterns with pollen counts and airborne fungi

**Fig. 5:**
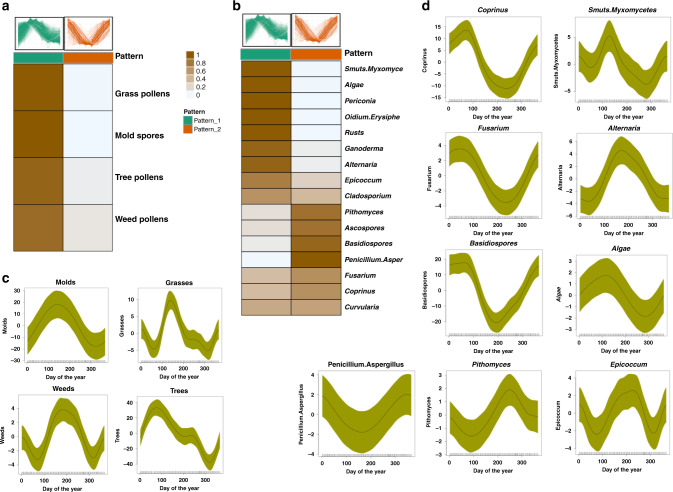
Airborne pollens and fungi correlate with omics seasonal patterns. Correlation of (**a**) pollen counts (tree pollens, weed pollens, mold spores, grass pollens), and (**b**) airborne fungi with the two omics seasonal patterns. The heatmap shows cluster membership values (which are on a scale of 0 and 1) based on existing cluster centroids (omics pattern one is shown in green color and omics patterns two are shown in orange color). Seasonal patterns of tree pollens, weed pollens, mold spores and grass pollens are shown in (**c**) and seasonal patterns of specific airborne fungi are shown in (**d**). The *Y*-axis shows the days of the year and the *X*-axis shows normalized pollen counts or normalized airborne fungi counts. The shaded area represents 95% confidence bounds computed as ±1.96 standard deviation of model coefficients. Standard deviations were derived from a maximum likelihood fit.

To evaluate the relationship between regional pollen exposure and omics seasonal patterns, we calculated GAMM seasonal coefficients for pollen counts (tree pollens, weed pollens, mold spores, grass pollens), as well as 23 airborne fungi collected from the San Francisco Bay area (Los Altos Hills, California) (Fig. 5a, b, Supplementary Fig. 9; Supplementary Table 4; Supplementary Data 10). We then correlated pollen count patterns with the two major omics seasonal patterns (Fig. 5a, b). We observed that total counts for grass pollens, mold spores, tree pollens, and weed pollens correlate with omics seasonal pattern one, where the peak allergy season starts around early spring (Fig. 5a). Total tree pollen counts peaks in early springtime, followed by total grass pollen counts and total mold spores counts that peak in late spring. These peaks also correlate with the allergy season in the San Francisco bay area that extends from March through June. There is also a surge in weed pollen that peaks in around mid-summer (Fig. 5a). Figure 5b shows the correlation of specific airborne fungi with omics seasonal pattern one and two. The individual seasonal patterns of airborne fungi are shown in Fig. 5b; Supplementary Fig. 9. Airborne fungi show seasonal changes and correlate with both omics seasonal pattern one and two (Fig. 5b). Fungal spores that peak in early spring/early summer and correlate with omics seasonal pattern one are *Rusts*, *Smuts*/*Myxomycetes, Algae, Oidium/Erysiphe, Periconia* and *Ganoderma*. Fungal spores that peak in late fall/winter are *Penicillium/Aspergillus, Ascospores, Basidiospores* and *Pithomyces.* Thus, pollen and spore counts associate with the major patterns and may be contributors to driving these patterns.

### Seasonal effects in insulin resistant and insulin sensitive individuals

**Fig. 6:**
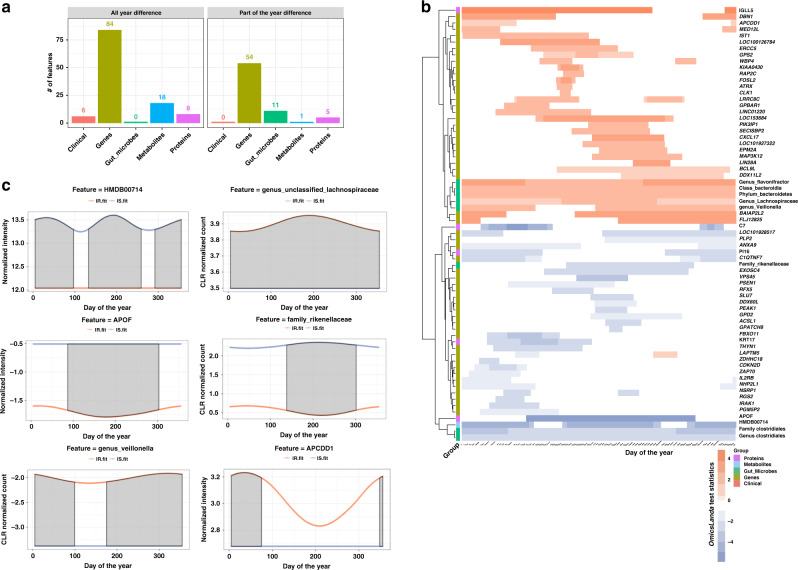
IR and IS responses to seasons. **a** 187 analytes show seasonality difference between IR and IS, of which 116 features exhibit global differences between IR and IS throughout the entire year and 71 features show the statistical significance during part of the year (*OmicsLonDA* permutation test *p* ≤ 0.05). **b** Examples of omics analytes that show significant differential abundance/expression between IR and IS subjects across the year. For each analyte, the orange curve represents fitted curve for IR, and dark blue curve represents the fitted curve for IS subjects. Gray shaded area between the two curves highlights time intervals when the corresponding feature is significantly different between the two groups (IR and IS). **c** Significant time intervals of analytes that show differences between IR and IS in part of the year. Each row represents an analyte, and the shaded area represents the time of the year that each corresponding feature shows a significant difference between IR (orange) and IS (blue) based on *OmicsLonDA* test statistics of each time interval.

Whether seasonal patterns are distinct between people with different diseases has not been investigated. Since the participants in our study have been well characterized as either insulin resistant (IR) or insulin sensitive (IS), we examined the seasonal differences of biomolecules and microbes in these two groups. Our aim is to identify time intervals of omics features that show differences between IR and IS groups either in part or all over the entire year. For this purpose, we used the multiomics profiles from 66 subjects who were classified as either IR (35 subjects) or IS (31 subjects) based on steady-state plasma glucose (SSPG) measurements (Supplementary Table 3). We utilized our recently developed longitudinal analysis method, *OmicsLonDA*^31^ (see “Methods”), to identify the time intervals where differences are observed (Supplementary Data 11 and 12). Of 11,184 analytes examined (all omics analytes combined), there were 187 omics analytes (138 genes, 13 proteins, 19 metabolites, 6 clinical markers, and 11 microbial taxa) that showed significant (*p* ≤ 0.05) seasonal differences between IR and IS (Fig. 6a). Of 187 significant analytes, we identified 71 that showed statistical significance in part of the year (Fig. 6c), whereas the remaining 116 features exhibit a global difference between IR and IS across the entire year. More specifically, at the microbiome level, *Veillonella* has a higher abundance in IR than IS throughout the year except mid-March until late June (Fig. 6b). The family *Rikenellaceae* from phylum *Bacteroides* is enriched in IS between mid-April until the end of October (Fig. 5b). The *Lachnospiraceae* family and *Flavonifractor* genus are examples of microbes that show a significant increase in IR than IS over the entire year (Fig. 6b). On the transcriptome level, 138 genes show differential seasonal effects (Supplementary Data 12). Among those genes are *APCDD1*, *PL2*, *GPS2*, and *EXOSC4*. *APCDD1* which showed higher expression in IR than IS during December until March (Fig. 6b). At the proteome level, APOF, C7, KRT17, and PI16 show significantly higher expression in IS than IR in part of the year, whereas IGLL5 showed a significant increase in IR than IS (Supplementary Data 12). At the metabolome level, our results indicated that 18 metabolites show significant changes between IR and IS across the entire year. It is of note that only Hippuric Acid (HMBD00714) is in higher abundance in IS than IR all year, except during April and October **(**Fig. 6b**)**. Among metabolites that have higher expression in IR are L-Octanoylcarnitine, Adrenic acid, Dihomo-gamma-linolenic acid, gamma-Linolenic acid, Eicosadienoic acid, 2-Octanoylcarnitine, 3, 5-Tetradecadiencarnitine, and Linoleic acid. Butyric acid, l-Malic acid, Cholic acid, N6-Trimethyl-l-lysine, Phenylacetylglutamine, Cinnamoylglycine, p-Cresol glucuronide, and 20-Dihydroxy Eicosanoic acid. Furthermore, three clinical markers, Neutrophils absolute count, Platelet count, and Triglycerides (TG) level, were found to be higher in IR than IS across the year. Overall, these results reveal that there are extensive differences between IR and IS participants.

### Lifestyle factors

We analyzed lifestyle factors that may have seasonal effects, such as dietary habits and physical activities^32,33^. Dietary habits data were collected every 3 months using the Dietary Targets Monitor survey^34^ (see “Methods”). The survey asks about the frequency of consumption of a variety of foods including fish, meat, cheese, fruits and vegetables, starchy foods as well as sweets, and savory snacks and comprises a total of 25 different food categories (see “Methods”, Supplementary Data 15 and 16). None of these food categories were significantly different between IR and IS groups throughout the year (One-way ANOVA with random blocks, *P*-value > 0.05, Supplementary Table 5, Supplementary Fig. 10). In our analysis we used subject ID as a random effect to account for different numbers of samples per subject. On the other hand, physical activity measured in total metabolic equivalent of task (MET) is significantly different between the IR and the IS groups in February, May, June, and August (*P*-value = 0.01787, Supplementary Fig. 11). However, a post-hoc analysis of all the omics features that were identified to be significantly different between the IR and the IS groups, are not associated with the physical activity.

## Discussion

In order to understand the molecular and microbial changes that occur during seasonal variations, we profiled 105 participants in the San Francisco bay area over 4 years. This study is unique in three respects. First, it performs intensive longitudinal profiling of 105 individuals from the same region for up to nearly 4 years across (105 individuals). Second, it holistically captures different types of omics data from the host (transcriptome, proteome, metabolome, cytokinome, clinical health data), as well as nasal and gut microbiota thereby enabling a deep profiling of human physiology and function. Third, it integrates data from environmental factors such as meteorological data (e.g. air temperature, solar radiation) and airborne pollen counts from the San Francisco Bay area. As a result of this comprehensive approach, our study discovered the seasonal changes of over 1000 molecules and microbes and identified two major biological seasonal patterns that correlate with early spring and late fall/early winter in the San Francisco bay area. The two major seasonal patterns of host molecules and microbes are relevant to human health and disease based on the biological processes in which they participate.

We have discovered omics features and bacteria taxa and genes with seasonality trends. Among clinical markers, HbA1c was found to peak in spring/summer and declines in winter. HbA1c is a common marker for T2D; it reflects the average blood glucose over the past 100 days^19^. Previous studies have compared the level of blood glucose in winter to summer, and report either higher blood glucose level in winter than in summer^35^, or no significant difference^36^. HbA1c also has been reported to be higher in winter in diabetic patients^37,38^. However, these studies only sampled patients in winter and summer (not throughout the year), and they were only conducted on diabetic patients. Higher HbA1c in Spring is likely explained by reduced physical activity in winter and a delayed effect due to the average nature of the measurement.

*PER1*, the primary circadian pacemaker in the mammalian brain, also shows a complex seasonal pattern with highest expression in spring. *PER1* belongs to a family of genes responsible for the circadian rhythms. There is emerging evidence suggesting that the deregulation of *PER1* plays an important role in the development of mammalian cancer^39^. Our data showed that *PER1* peaks in spring; interestingly, seasonal variation studies in tumor stage at the time of diagnosis shows spring as the season with the highest proportion of localized tumors^40^, indicating that tumor stage correlates with seasonal activity. *C2, C6, IL5, SIGLEC15,* and *IL1RAP* have roles in immune responses. *SIGLEC15* and *IL1RAP* peak in late spring/early summer, consistent with their role in allergies which often peaks in spring and early summer, and *IP10, C2* and *C6* peak in late fall/winter consistent with their role in fighting infections which often occur during the late fall/winter.

Consistent with the molecular patterns, we also found seasonal association of biological pathways that are relevant to human physiology and disease. The immune responses such as the complement system, acute phase response and IL12 signaling pathways are highest in fall/winter, as expected for when respiratory viral infections are common (Fisher’s exact test *p*-value ≤ 0.05). Pathways related to knee and joint inflammation such as osteoarthritis and rheumatoid arthritis pathways are elevated in spring/summer (Fisher’s exact test *p*-value ≤ 0.05). Molecular signatures associated with neurological and behavioral pathways also show seasonal patterns. Schizophrenia spectrum disorder, seizures, and sleep disorder are enriched at late spring/early summer (omics seasonal pattern one). Such seasonal patterns have been previously shown to be associated with several aspects of human brain functions such as mood and cognition, and influence many neurological and psychiatric illnesses, and the incidence of schizophrenia^41^.

Molecular signatures associated with hypertension, blood pressure and cardiovascular diseases correlate with the late spring/early summer (seasonal pattern one) and are consistent with previous studies demonstrating that blood pressure (BP) is higher in winter^6,42^. As above, the late Spring/early summer may reflect the end of a period of reduced activity. Interestingly, Sjögren’s syndrome (a chronic autoimmune disorder characterized by lymphocytic infiltration and malfunction of the exocrine glands, causing dry mouth and eyes)^43^, inflammation of the myocardium, volume of urine, and esterification of cholesterol are enriched during late fall/early winter (seasonal pattern two (Fisher’s exact test *p*-value ≤ 0.05)) (Fig. 2g).

We also find acne is associated with seasonal pattern two, consistent with previous reports^44^. Demographic studies have shown that acne worsens in winter and improves in summer^44^. Cold air temperature and low humidity in winter increase skin permeability, epidermal thickening, and stimulate inflammatory mediator production, which leads to acne worsening in winter^45^. Our data show that azelaic acid, SELL (Leukocyte-Endothelial Cell Adhesion Molecule 1), APOA1 (Apolipoprotein A1) and RBP4 (Retinol Binding Protein 4) are the drivers of seasonal responses contributing to acne pathophysiology. Azelaic acid (dicarboxylic acids) and retinol are currently used to treat mild to moderate acne^41,46^. Our data therefore support seasonal adjustment for acne treatment. Altogether, our results provide molecular signatures for a variety of seasonal conditions and processes, and provide molecular and biological insights into these processes as well as potential biomarkers for monitoring and treating increased seasonal risk for a variety of diseases.

We also analyzed the microbiome in detail and discovered a clear seasonal trend of nasal and gut microbiome composition (Fig. 4c). We observed significantly higher diversity for gut microbial taxa during February–April and significantly lower diversity in September–October (*GAMM* model likelihood ratio test *p*-value ≤ 0.05) (Fig. 4c). We also observed significantly lower diversity for nasal microbial taxa during spring, which coincides with allergy season in the San Francisco bay area (*GAMM* model likelihood ratio test *p*-value ≤ 0.05) (Fig. 4c). Interestingly, recent studies also indicate that asthma may be associated with decreased nasal and gut microbiome diversity^47,48^, and our seasonality finding supports this association, as it coincides with the annual peak in asthma exacerbations^49^.

Moreover, there are more microbial taxa with seasonal effects in nasal samples (26 microbial taxa) compared to the gut (four microbial taxa) (Supplementary Data 2). This may be due to the more diverse seasonal exposure of the nasal environment as compared to the gut which is heavily influenced by food; although there are seasonal patterns of food consumption, our results suggest food diversity may be less seasonally diverse than airborne exposures which are known to be highly dynamic at the personal level^50^. A notable nasal microbial taxonomy with high degree of seasonality variation is *Staphylococcus aureus*, a key pathogenic bacterium in chronic rhinosinusitis with nasal polyps, that appears to be more abundant in September-October period (Supplementary Fig. 8a). Other notable bacterial taxa include *Porphyromonas* genus, *Bacteroidia* class, *Actinobacteria* class and *Pasteurellaceae* family which correlate with omics seasonal pattern one, while *Firmicutes* phylum, *Bacilli* class, *Dolosigranulum* genus, *Corynebacterium* genus correlate with omics seasonal pattern two (Fig. 4b). Furthermore, gut microbial taxa *Holdemania* genus, *Ruminococcaceae* genus, and *Oscillibacter* genus, as well as *Firmicutes* phylum, show significant (*P* ≤ 0.05) seasonal components. It is of note that the gut *Firmicutes* and the nasal *Firmicutes* show different seasonal patterns (Supplementary Fig. 8b). While the gut genus *Firmicutes* is in higher abundance through March to April and in lower abundance through August to September, the nasal phylum *Firmicutes* peaks through April to September and declines in winter (Supplementary Fig. 8b).

We discovered that the two omics seasonal patterns correlate with airborne pollen counts and fungi in the San Francisco Bay area. This correlation may suggest the role of tree pollens, grass pollens, weed pollens and mold spores in contributing to the regulation/dysregulation of biochemical and immunological pathways segregated with the omics seasonal patterns. These patterns are also in alignment with allergy seasons (e.g. allergic rhinitis) in the San Francisco Bay area. In general, our data indicate that tree pollens are the cause of allergies in early Spring in the Bay area, while grass pollens are the cause of allergies in late Spring/Early Summer along with mold spores. Our data provide information in regard to the onset, duration and severity of the pollen season in the Bay area.

We examined differences in seasonal patterns among IS and IR individuals. We identified 187 omics features and microbial taxa which show significantly seasonal differences between the IR and IS groups. For instance, *Veillonella* has a higher abundance in IR than IS individuals throughout the year, except mid-March until late June (Fig. 5b). Previous studies have shown that *Veillonella* genus, a gram-negative bacteria known for its lactate fermentation abilities, is associated with IR^51^. Here we demonstrate these differences are strongest all year long except in Spring (day 100–day 175) (Fig. 5b). In contrast, family *Rikenellaceae* from phylum *Bacteroides* is enriched in IS between mid-April to the end of October (Fig. 5b). *Rikenellaceae* is linked with enhanced insulin sensitivity in mice^52^. The *Lachnospiraceae* family contains further examples of microbes that show a significant enrichment in IR across the year (Fig. 5b). *Lachnospiraceae* is reported to be associated with T2D in human^53^, and the colonization of the gut by a *Lachnospiraceae* bacterium contributes to the development of diabetes in obese germ-free mice^54^. Another notable molecule with seasonal differences between IR and IS groups is *APCDD1*, which showed higher expression in IR than IS during December until March (Fig. 5b). *APCDD1* inhibits the Wnt signaling pathway^55^, and the downregulation of the canonical Wnt signaling pathway is associated with hypertension^56^, which is also more prevalent in winter^57^. As has been demonstrated previously^58,59^, three clinical markers: neutrophil absolute count, platelet count, and triglyceride (TG) level were found to be higher in the IR group throughout the year. However, the relationship of increased platelet counts to insulin resistance needs further validation.

Several omics analytes that we identified in our study are linked with hibernation. Hibernation is a dynamic phenotype maximizing energy storage during periods of low resource availability and metabolic depression^60^. Supplemental figure 12 shows molecules from our study that are known to change during periods of hibernation in mammals^60,61^. For example, serpin peptidase inhibitor (SERPINC1) protein is shown to be overexpressed in late torpor compared to early arousal time in hibernating arctic ground squirrels^62^. Our data also show overexpression of SERPINC1 in winter compared to summer (Supplementary Fig. 12).

All together, these deep multiomics measurements enable us to: 1. Identify extensive seasonal variations across omics molecules and microbiomes; 2. Discover biological pathways that drive seasonal responses for human diseases such as cardiovascular and hypertension disorders; and 3. Detect two basic omics seasonal patterns that correlate with late spring and late fall/early winters in the San Francisco bay area. It is of note that from the climate perspective, there are mainly two seasons in the San Francisco Peninsula: a long and moderately warm and dry summer, and a mild winter when it rains. The two major human biological seasonal patterns are likely influenced by these environmental conditions, which likely impact environmental exposures as well as lifestyle. We expect that late spring/early summer (pattern one) reflects a period at the end of less physical activity and the onset of heavy pollen count; late fall/early winter likely reflects a period of high RVI. Importantly, these results have direct implications in disease risk monitoring and for discovering health biomarkers, given our finding that these markers carry seasonal variation which needs to be measured when interpreting disease risk. It is of note that our data do not capture changes associated with solar diurnal cycle and dark–light cycle as we collected one sample per each visit from fasted participants. We tried to minimize the effect of solar diurnal cycle on our seasonal changes assessment by taking samples at the same time for each fasted participant (7–10 am) for every visit over the course of 4 years. However, time of sample collection is different relative to the dark–light cycle that is known to have an impact on the phase of sleep and human physiology^63^. Therefore, the seasonal differences that we report in this study may be affected by different phases of the seasonal dark–light cycle. We also suggest researchers consider photoperiodism and other recurring patterns (e.g. the menstrual cycle) in their study design for future efforts. Finally, since every geographical location has its unique climate conditions, our approach can be applied to any geographical location around the planet to capture the seasonal human biology associated with these locations.

## Methods

### IRB and informed consent

This study complies with all relevant ethical regulations and has been approved by the Stanford University Institutional Review Board. All participants provided written informed consent and were enrolled as part integrative personalized omics profiling project at Stanford. This study conducted under research study protocol IRB 23602 and all research participants were studied after an overnight fast at the Stanford Clinical and Translational Research Unit (CTRU). Participants survey information were managed using REDCap electronic data capture tools hosted at Stanford University.

### Multiomics experiments and data preparation

Multiomics data of host and microbial samples used in this study are taken from our integrative personalized omics profiling project^17^. A brief description of these data is as follows. RNA-seq: the transcriptome was evaluated by RNA sequencing (RNA-seq) from bulk PBMCs. The RNA libraries were constructed using the TruSeq Stranded total RNA LT/HT Sample Prep Kit (Illumina). The TopHat package was used to align the reads to the human reference genome (hg19). HTseq and DESEQ2 were used for quantification and analysis. Custom scripts in R and Python were used for downstream analyses. For data preprocessing, we first removed the genes with average read counts over all samples smaller than 1. Then the samples with average read counts over all filtered genes smaller than 0.5 are filtered out. After this, we have 890 samples with the expression level data of 10142 genes. Microbiome Sampling and analysis: stool and nasal microbiome sampling were collected according to the Human Microbiome Project–Core Microbiome Sampling Protocol A (hmpdacc.org) and (HMP Protocol #07-001, v12.0). Targeted rRNA gene amplification and sequencing was carried out by 16S (Bacterial) rRNA gene amplification V1-V3 of 16S using primers 27F and 534R (27F:5′-AGAGTTTGATCCTGGCTCAG-3′ and 534R: 5′- ATTACCGCGGCTGCTGG-3′).

*Untargeted Metabolomics by LC-MS*: Metabolic extracts from plasma were analyzed using HILIC. Metabolic extracts from all samples were prepared in a randomized order. Only metabolic features present in >33% of the samples were kept for further analysis and missing values were imputed using the k-nearest neighbors’ method. Inter- and intra-batch variation was corrected using the LOESS normalization method on QC injected repetitively along the batches. After normalization, samples from the same individual tend to cluster together. A total of 722 metabolites were measured using our metabolite profiling platform among which 431 were identified by matching retention time and fragmentation spectra to authentic standards or by comparing fragmentation spectra to public repositories. Proteomics (SWATH-Mass Spectroscopy) Tryptic peptides of plasma samples were obtained from NanoLC™ 425 System (SCIEX). Peak groups from individual runs were statistically scored with pyProphet and all runs were aligned using TRIC9 to produce a final data matrix. Luminex Assays: levels of circulating cytokines, chemokines and growth factors in the blood were measured using a 63-plex Luminex antibody-conjugated bead capture assay (Affymetrix) that has been extensively characterized and benchmarked by the Stanford Human Immune Monitoring Center (HIMC). The full list of cytokines, chemokines and growth factors are shown in Supplementary Data 5 (Supplementary Data 5). Clinical laboratory tests: all visits were intensively characterized by 51 clinical laboratory tests (Supplementary Data 6).

*Modified Insulin Suppression test*: Steady-state plasma glucose was measured using the modified insulin suppression test^20^, which consists of an overnight fast followed by an 180-min infusion of octreotide (0.27 μg m^−2^ min^−1^), insulin (0.25 μg m^−2^ min^−1^) and glucose (240 μg m^−2^ min^−1^). Blood draws were then taken at minutes 150, 160, 170, and 180. The oximetric method was used to determine blood glucose and SSPG was determined by taking the mean of the four measurements.

*Meteorological measurements*: We obtained historical meteorological parameters on visit days that we have samples collected from our cohort. We queried the synoptic portal (https://synopticdata.com/) to retrieve the Meteorological measurements from Menlo Park station (Menlo Park, CA). The majority of our subjects are residents of the San Francisco Bay Area (Supplementary Data 1, Supplementary Fig. 9). The collected parameters include air temperature (Celsius), humidity (%), solar radiation (W/m^2^), pressure (k Pascal), wind speed (m/sec), and precipitation (mm) (Supplementary Data 8).

### Airborne pollen and mold counts

Pollen and airborne fungi counts have been collected from the American Academy of Allergy, Asthma, and Immunology (AAAAI) station located at Los Altos Hills, California, USA. The counts are collected on a weekly basis between 2014 and 2016. Pollen counts are measured from silicone-greased glass slides taken from the Burkard Collector in pollen grains or mold spores per cubic meter of air per 24 h. The counts are done manually. The slide is stained with four drops of Calberla’s solution, which is a red dye of basic fuchsin crystals, and ethanol, and a little bit of glycerin. A cover slip is put on the slide, it is put on a microscope and a sweep is made from one end of the cover slip to the other. All pollens are either identified as grass, specific trees, or weeds, but there are always a number of pollen grains which are unidentified. Also, it is worth mentioning that pollen and mold counts can only be generalized to all locations in a very imprecise manner. If a location has lots of oak trees around, similar to where the station is located, the oak pollen count will be higher. Similarly, if there are acacia trees around, the acacia count will be higher, dissimilar to where the station is located. Pollen and airborne fungi data used in this study can be found at Supplementary Data 13 and 14.

### Physical activity and dietary habits

The International Physical Activity Questionnaire (IPAQ) short form, a validated instrument, was used to assess physical activity in participants every 3 months^34^. The iPAQ asks about days and minutes of vigorous, moderate and walking activity in the past week and it was scored per IPAQ protocol to estimate the total metabolic equivalent of task (MET) minutes expended. Dietary habits were similarly assessed every 3 months using the Dietary Targets Monitor which asks about the frequency of consumption of a variety of foods including fish, meat, cheese, fruits and vegetables, starchy foods as well as sweets, and savory snacks^64^. Dietary habits and IPAQ data can be found at Supplementary Data 15 and 16.

### Statistical analysis of seasonality modeling

In order to analyze the seasonality of longitudinal multiomics data of host and microbiome, we used generalized additive mixed model (*GAMM*) in *mgcv* package in R^63^. The gamm function in the *mgcv* package allows fitting smoothing terms (a cyclic cubic regression splines) to model seasonal time-series data. The cyclic cubic regression spline ensures that the measurement at the end of the year (day 365) is the same as the beginning of the year (day 1). We also considered subject’s ID as random effect, and subject’s BMI and health status (IR and IS) as covariates:

formula = y ~ IRIS + BMI + s(Time, bs = "cc"). The GAMM coefficients are extracted via gam$coefficients object in *mgcv* package. The P-values for smooth terms are extracted using summary(mod$gam), which is derived based on a likelihood ratio test^63^. Omics features with seasonality pattern are those with *GAMM* model likelihood ratio test *p*-value ≤ 0.05.

### Seasonal clustering analysis of omics feature

Omics features that have a *GAMM* model likelihood ratio test *p*-value ≤ 0.05 (Supplementary Data 2) (as described above), were selected for clustering analysis. We then derived the GAMM coefficients for the significant features (*GAMM* model likelihood ratio test p-value ≤ 0.05) for every type of ome (Supplementary Data 7). Subsequently, we performed Elbow and Silhouette criteria to identify the optimum number of clusters based on C-means clustering for every single ome as well as all omes combined. The silhouette coefficient estimates the average distance between clusters. We also conducted the principal component analysis (PCA) on the GAMM coefficients for all the omics analytes and Fig. 2a shows the scatter plot of the first two PCs, colored by cluster numbers (cluster 1 and 2) and 95% confidence interval. Each omics analyte was normalized to have the same variance and the first two PCs explain most of the variance (93%). Having identified the best number of clusters (*k* = 2 in our case; Fig. 2a), we then conducted fuzzy C-means pattern recognition clustering on *GAMM* coefficients using the Mfuzz package in R^65^. We performed fuzzy C-means pattern recognition clustering on every single ome (transcriptome, proteome, metabolome and cytokines) as well as all combined.

### Correlation of omics seasonal patterns

In order to measure how well the gut and nasal microbiome, the clinical lab tests and the meteorological measurements fits the existing two omics seasonal patterns, we calculated cluster membership values (which are on a scale of 0 and 1) for new data (e.g., microbiome, clinical lab tests, meteorological measurements) based on existing cluster centroids and fuzzification parameters using *membership* function in *Mfuzz* package in R^65^ (Supplementary Data 9). We used *ggplot2* in R for visualization of the membership scores.

### Ingenuity canonical pathway and disease analysis

We applied ingenuity pathway analysis (IPA^®^, QIAGEN Inc.) to search for enriched pathways and diseases in our list of’omics molecules that carry a significant seasonal component. For integrated canonical pathway analysis as well as disease enrichment analysis, the significant transcripts, proteins, metabolites and cytokines were combined and used as an input file. The IPA enrichment algorithm uses the ‘enrichment’ score based on Fisher’s exact test *p*-value. The p-value represents the significance of the overlap between observed and predicted regulated molecules. We performed this analysis separately for omics molecules from seasonal pattern one and seasonal pattern two. We used *ggplot2* package in R to visualize the differences between the two seasonal patterns.

### IR and IS longitudinal analysis

We utilized our recently developed longitudinal analysis method, *OmicsLonDA* (https://bioconductor.org/packages/OmicsLonDA/), to find the time intervals of differentially abundant/expressed omics features between IR and IS. *OmicsLonDA* is an extension of *MetaLonDA*^66^ to account for correlated data, repeated measurements, and multiple covariates (continuous and/or categorical). For each feature *f* and for each group *k* (IR or IS), we used a generalized additive mixed model for modeling nonlinear time-series abundance/expression/intensity of omics features. Since we model the seasonality effect, we enforced such cyclicity in our model by using cyclic cubic spline, where the fitted model is continuous up to the second derivative. This ensures that the measurement on December 31st (day 365) is the same as January 1st (day 1). In our study, we accounted for the subject’s age, BMI, and health status as covariates, while subject’s ID as a random effect. Once the model is fitted, we did a hypothesis test on the coefficient of the time function *f(t)*. We then calculated the test statistic for each of the *T*-1 time intervals, where *T* is the number of timepoints (*T* = 365, in our case). We developed a studentized test statistic that quantifies the differences between the two splines for each time interval (Eq. (1)). The formula represents the area between the two splines for each time interval [*t*, *t* + 1] as shown in, where A^k1^[t, t + 1], and A^k2^[t, t + 1] denote the area under the spline curve from time *t* to *t* + 1 for group 1 (IR) and group 2 (IS), respectively, and SE represents the standard error.

1 $${\mathrm{testStatistic}}_{t,t + 1} = \frac{{A_{t,t + 1}^{k1} - A_{t,t + 1}^{k2}}}{{\sqrt {\left( {\frac{{SE_t^{k1} + SE_{t + 1}^{k1}}}{2}} \right)^2 + \left( {\frac{{SE_t^{k2} + SE_{t + 1}^{k2}}}{2}} \right)^2} }}.$$

Then, we performed a permutation procedure by permuting the sample group label *k*. The permutation was done *B* times (*B* = 1000, in our case), and after each permutation, we calculated the test statistics for the null hypothesis for each time interval. Subsequently, the p-value of each interval of the tested feature *f* was calculated using Eq. (2), where (*T*-1) denotes the number of time intervals, and I(.) is an indicator function.

2 $${{p{\hbox{-}}{\rm{value}}}}_{t,t + 1} = \frac{{\mathop {\sum }\nolimits_{j = 1}^{T - 1} \mathop {\sum }\nolimits_{b = 1}^B {\mathrm{I}}({\mathrm{testStatistic}}_{j,j + 1}^b \,> \, {\mathrm{testStatistic}}_{t,t + 1}^b)}}{{B \ast (T - 1)}}.$$

The *p*-value was then adjusted for multiple testing using Benjamini–Hochberg^67^ to control for the false discovery rate. For each feature *f*, significant time intervals are inferred as those with adjusted-*p*-value[*t*, *t*+1] < *α*/2, where *α* is the significance level (*α* = 0.05, in our case)^68^.

### Reporting summary

Further information on research design is available in the Nature Research Reporting Summary linked to this article.

## Supplementary information

Supplementary Information

Peer Review File

Reporting Summary

Description of Additional Supplementary Files

Supplementary Data 1-16

Supplementary Code 1-2

## Data Availability

All raw data used in this study are hosted on the NIH Human Microbiome 2 project site. (https://portal.hmpdacc.org). The processed data of host and microbiome data are available at https://figshare.com/articles/Multi_Omics_Seasonal_RData/12376508.

## Code availability

The code for *GAMM* model (Supplementary Code 1) and clustering (Supplementary Code 2) are available in supporting information files. The code for IR and IS analysis is available on https://github.com/aametwally/ipop_seasonal.
