# Supplementary material for: Deep longitudinal multiomics profiling reveals two biological seasonal patterns in California
Source: Nat Commun. 2020 Oct 1;11:4933. doi: 10.1038/s41467-020-18758-1 (PMC7529769; doi:10.1038/s41467-020-18758-1)
Supplement: Supplementary file 1 — Supplementary Information [file 41467_2020_18758_MOESM1_ESM.pdf]

Deep longitudinal multi-omics profiling reveals two biological seasonal patterns in California

M.Reza Sailani, *et al.*

Supplementary Figure 1

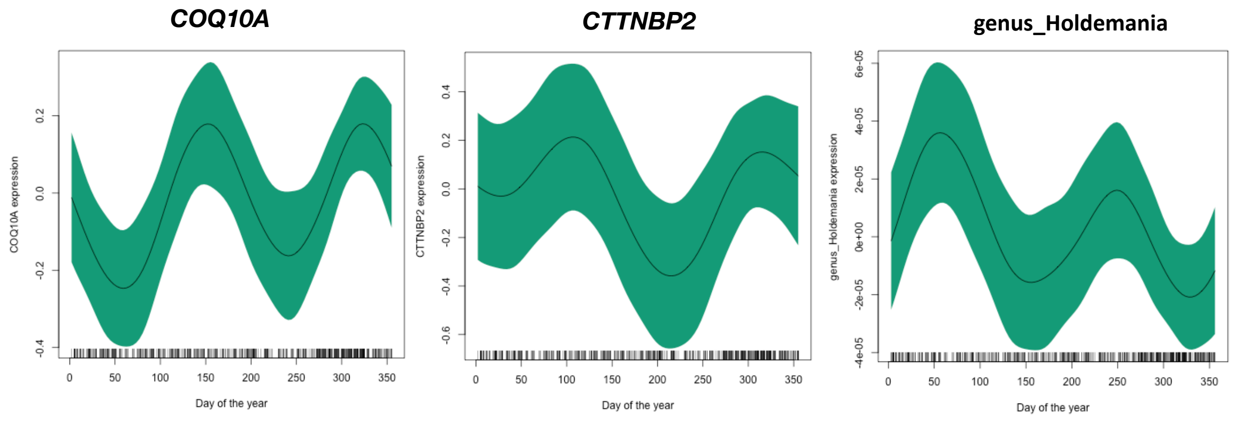

Supplementary Figure 1. Example of omics analytes that peaked twice or thrice per year. The shaded area represents 95% confidence bounds computed as +-1.96 standard deviation of model coefficients. Standard deviations were derived from a maximum likelihood fit.

Supplementary Figure 2

a.

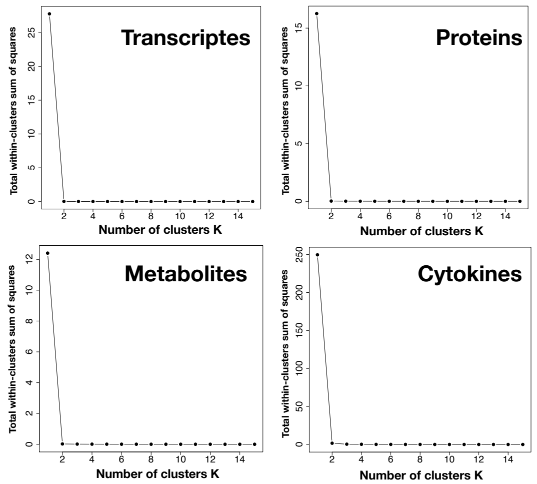

b.

All omes

Optimum number of clusters *k*=2

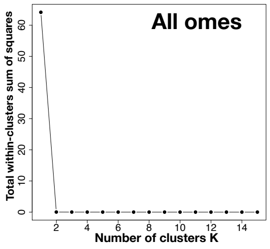

K	With-in-Sum-of-Squares
1	64.14135706
2	0.024522817
3	0.014521386
4	0.010667941
5	0.00847111
6	0.006989429
7	0.005913969
8	0.005122159
9	0.004565683
10	0.004048825
11	0.003669832
12	0.003497315
13	0.003171231
14	0.002833686
15	0.002634558

All omes (PCA analysis)

c.

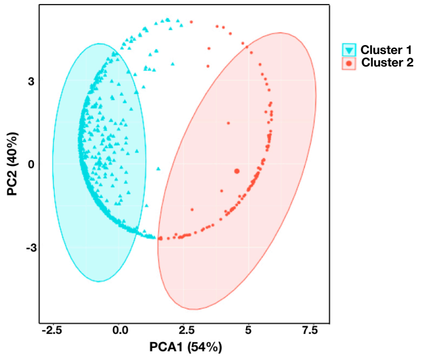

Supplementary Figure 2. a. Elbow plot of C-means clustering for identifying the optimal number of clusters for individual omes` (statistically significant features were used: GAMM model likelihood ratio test p-value≤0.05). For cytokines, all the 62 features were used. **b.** Elbow plot of C-means clustering for identifying the optimal number of clusters for all omes` combined. **c.** Scatter plot of the first two PCAs colored by cluster number are shown. The first two PCAs account for 93% of the total variances. All the analytes were normalized to have the same variance.

Supplementary Figure 3

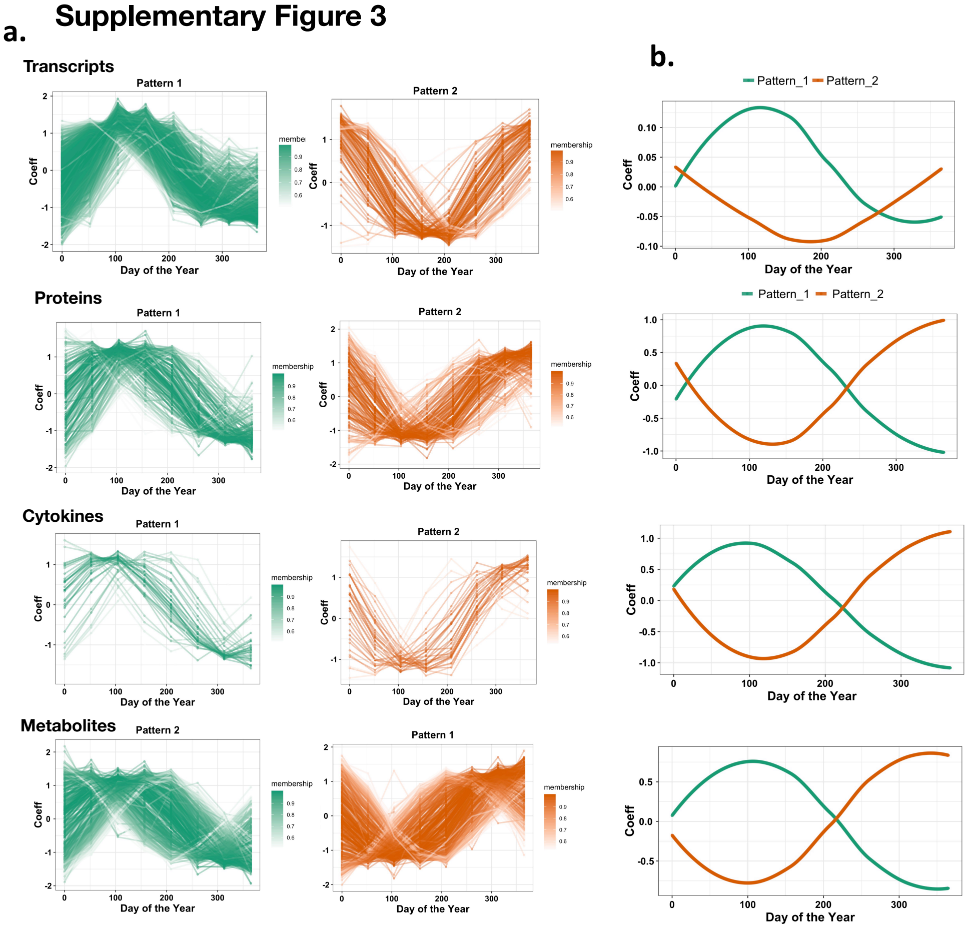

Supplementary Figure 3. **a.** Fuzzy C-means clustering of seasonal patterns for individual omes` (statistically significant features were used: *GAMM* model likelihood ratio test p-value≤0.05). For cytokines, all the 62 features were used. The X axis shows days of the year (1-365 days) and the Y axis shows normalized GAMM coefficients. **b.** Summarized patterns for individual omes`.

Supplementary Figure 4

The collagen genes super family.

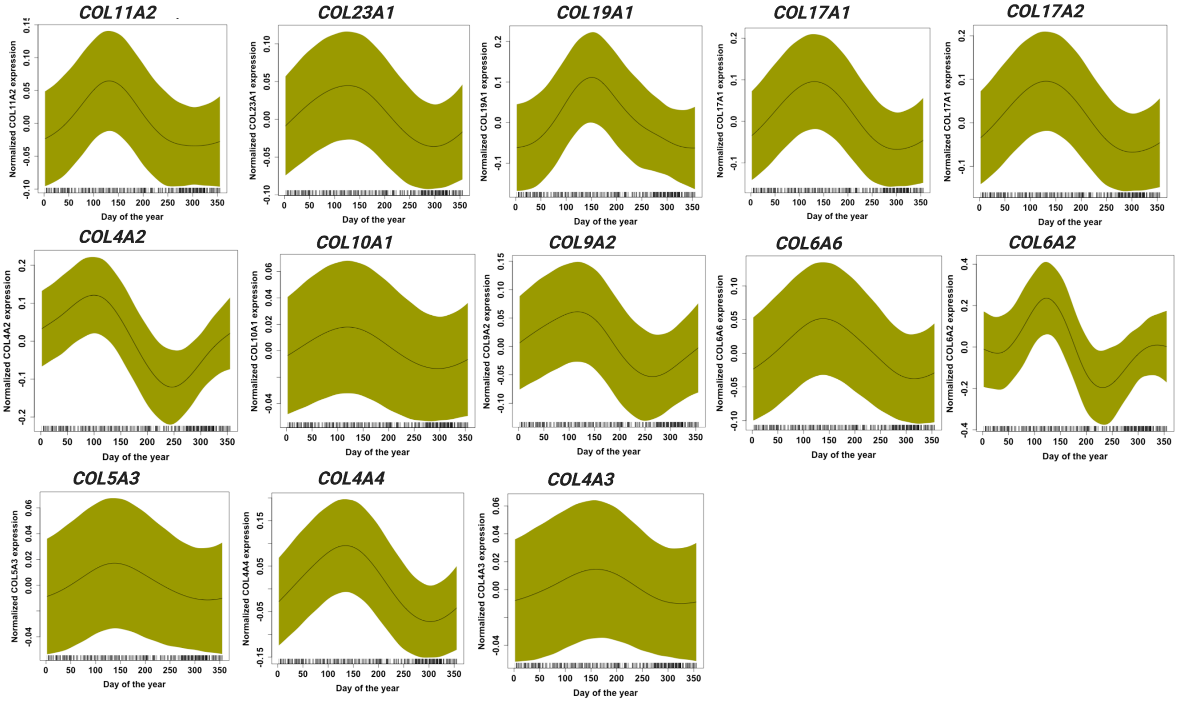

Supplementary Figure 4. Seasonal effects in collagen supergene family. The X-axis represents days of the year and Y-axis represents the normalized expression values. The shaded area represents 95% confidence bounds computed as +-1.96 standard deviation of model coefficients. Standard deviations were derived from a maximum likelihood fit.

Supplementary Figure 5

Acute phase response signaling pathway

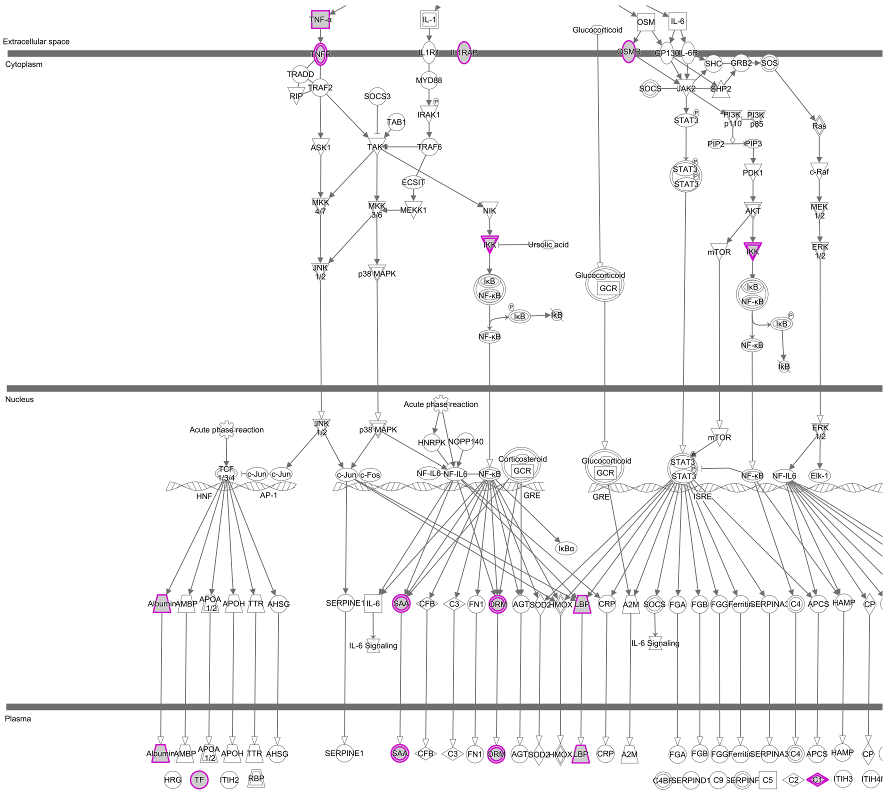

Supplementary Figure 5. Seasonal effects in acute phase response signaling pathway. Molecules highlighted in red show seasonal components.

Supplementary Figure 6

Complement system genes

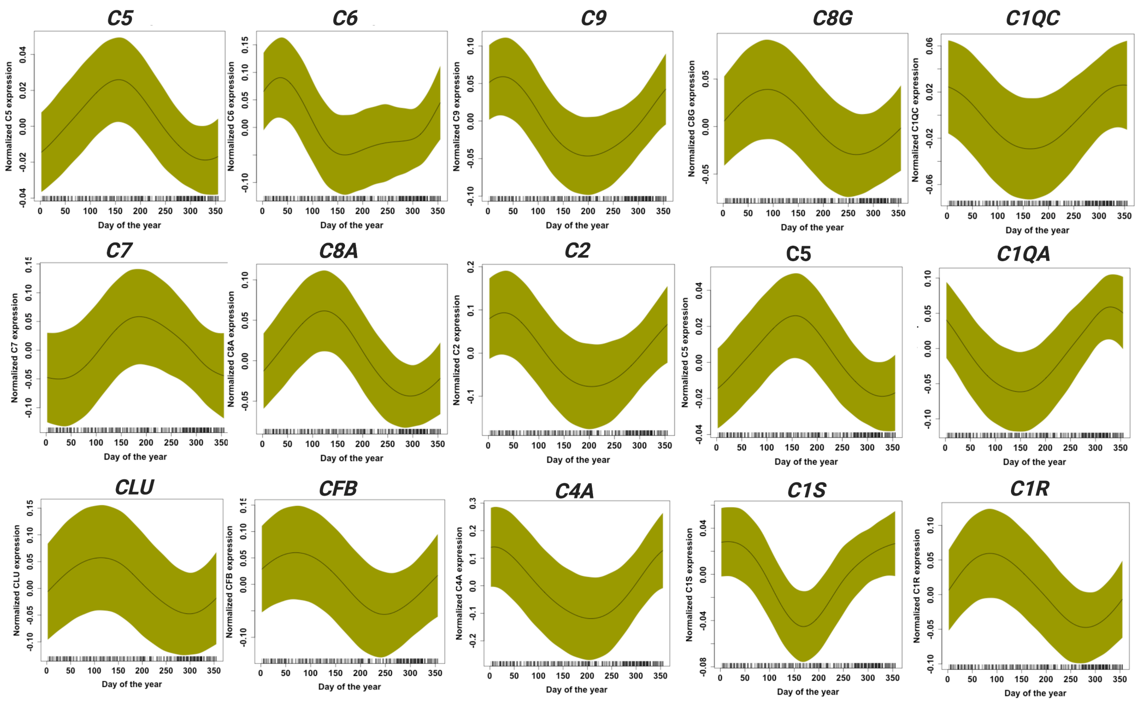

Supplementary Figure 6. Seasonal effects in Complement system genes. The X-axis represents days of the year and Y-axis represents the normalized expression values. The shaded area represents 95% confidence bounds computed as +-1.96 standard deviation of model coefficients. Standard deviations were derived from a maximum likelihood fit.

Supplementary Figure 7

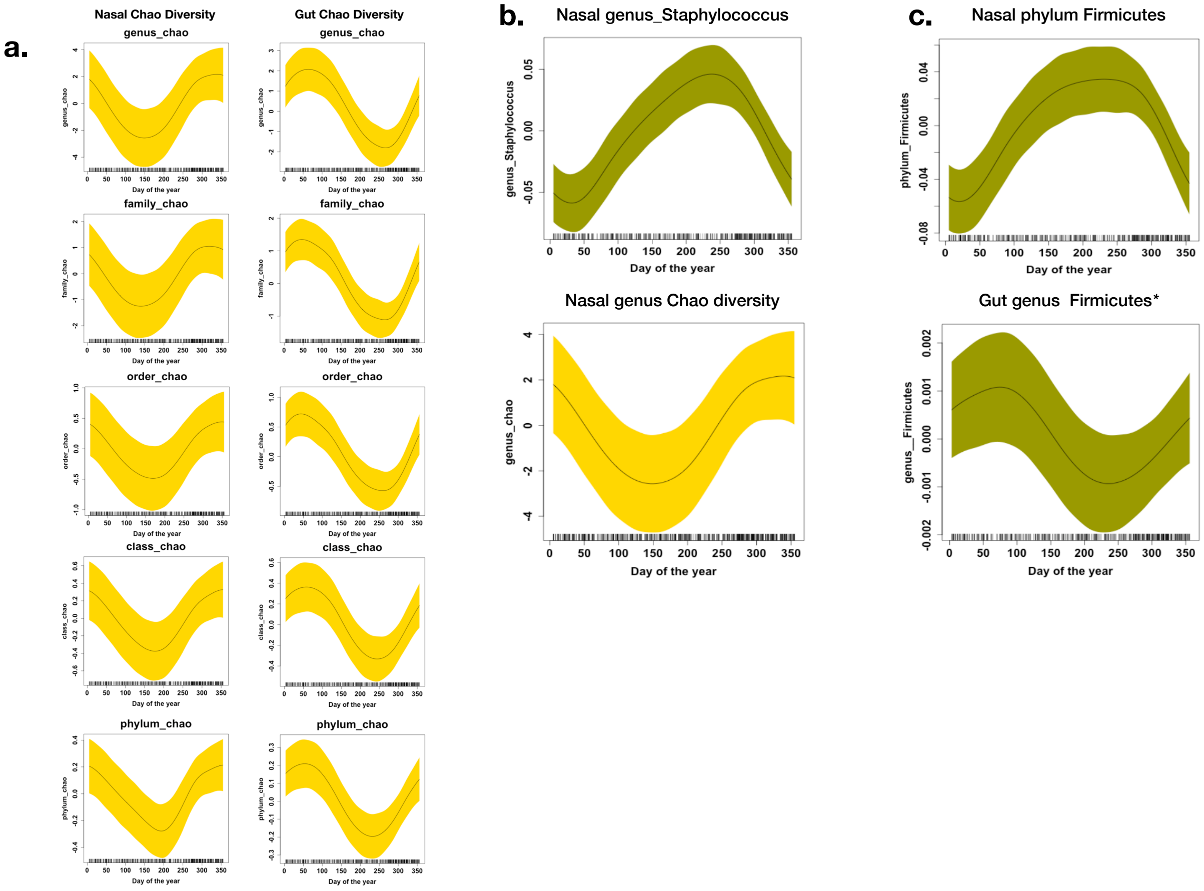

Supplementary Figure 7. **a.** Gut and nasal microbiome diversity scores (by Chao diversity) throughout the year. The Y axis shows the days of the year and X axis shows normalized Chao diversity scores. Diversity scores are shown for genus, family, order, class and phylum. **b.** The overall nasal microbial diversity anti-correlates with enrichment of Staphylococcus genus. c. The gut Firmicutes abundance anti-correlates with nasal Firmicutes. The shaded area represents 95% confidence bounds computed as +-1.96 standard deviation of model coefficients. Standard deviations were derived from a maximum likelihood fit.

Supplementary Figure 8

a.

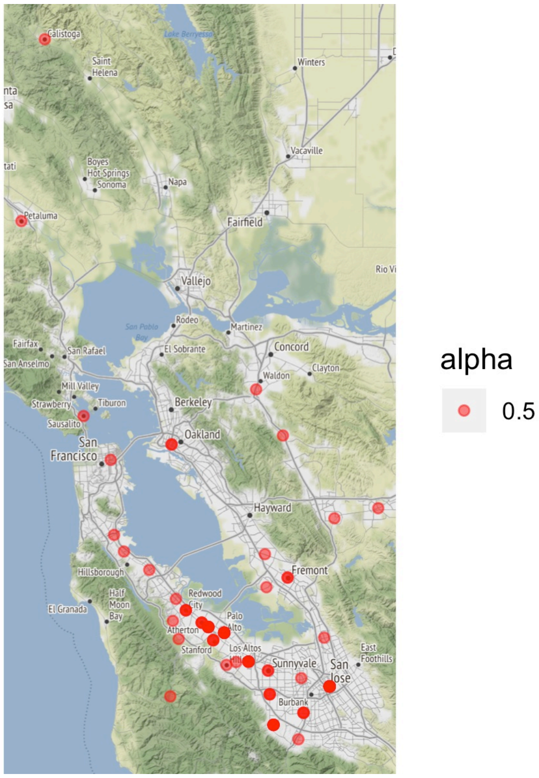

b.

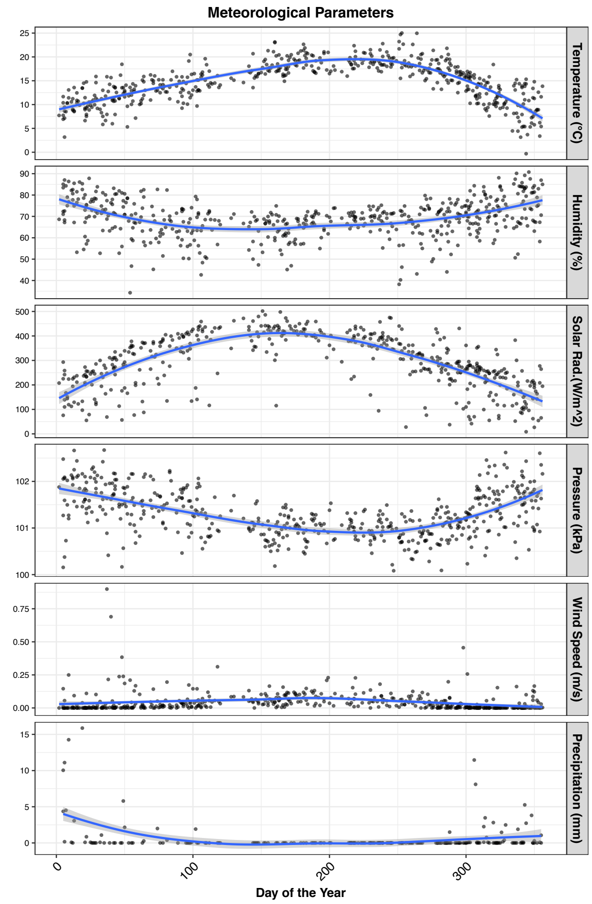

Supplementary Figure 8. a. Cohort residential locations in the San Francisco Bay Area. **b.** Meteorological parameters from the San Francisco bay area.

Supplementary Figure 9

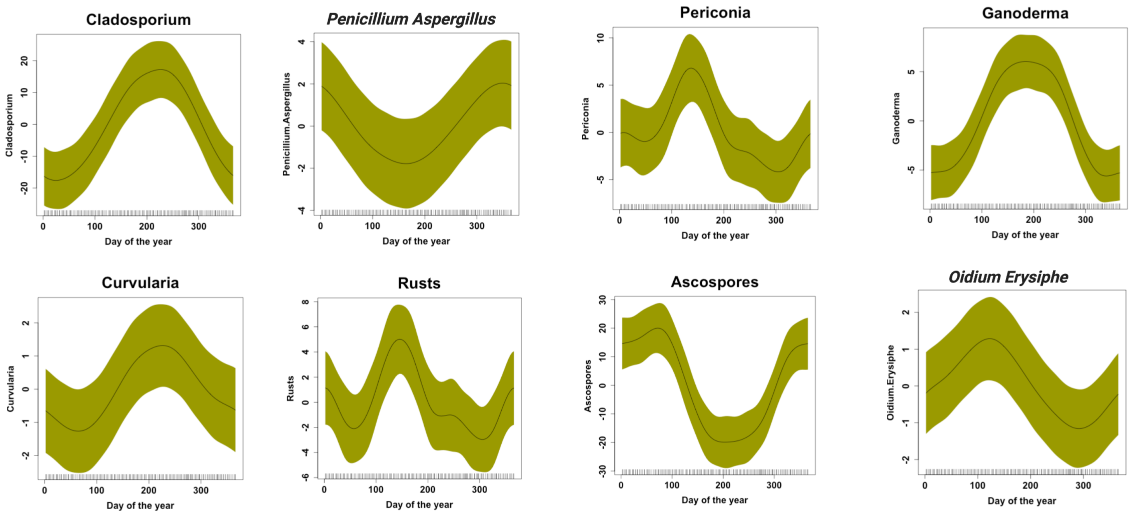

Supplementary Figure 9. Seasonal patterns of specific airborne fungi. The Y axis shows the days of the year and the X axis shows normalized airborne fungi counts. The shaded area represents 95% confidence bounds computed as +-1.96 standard deviation of model coefficients. Standard deviations were derived from a maximum likelihood fit.

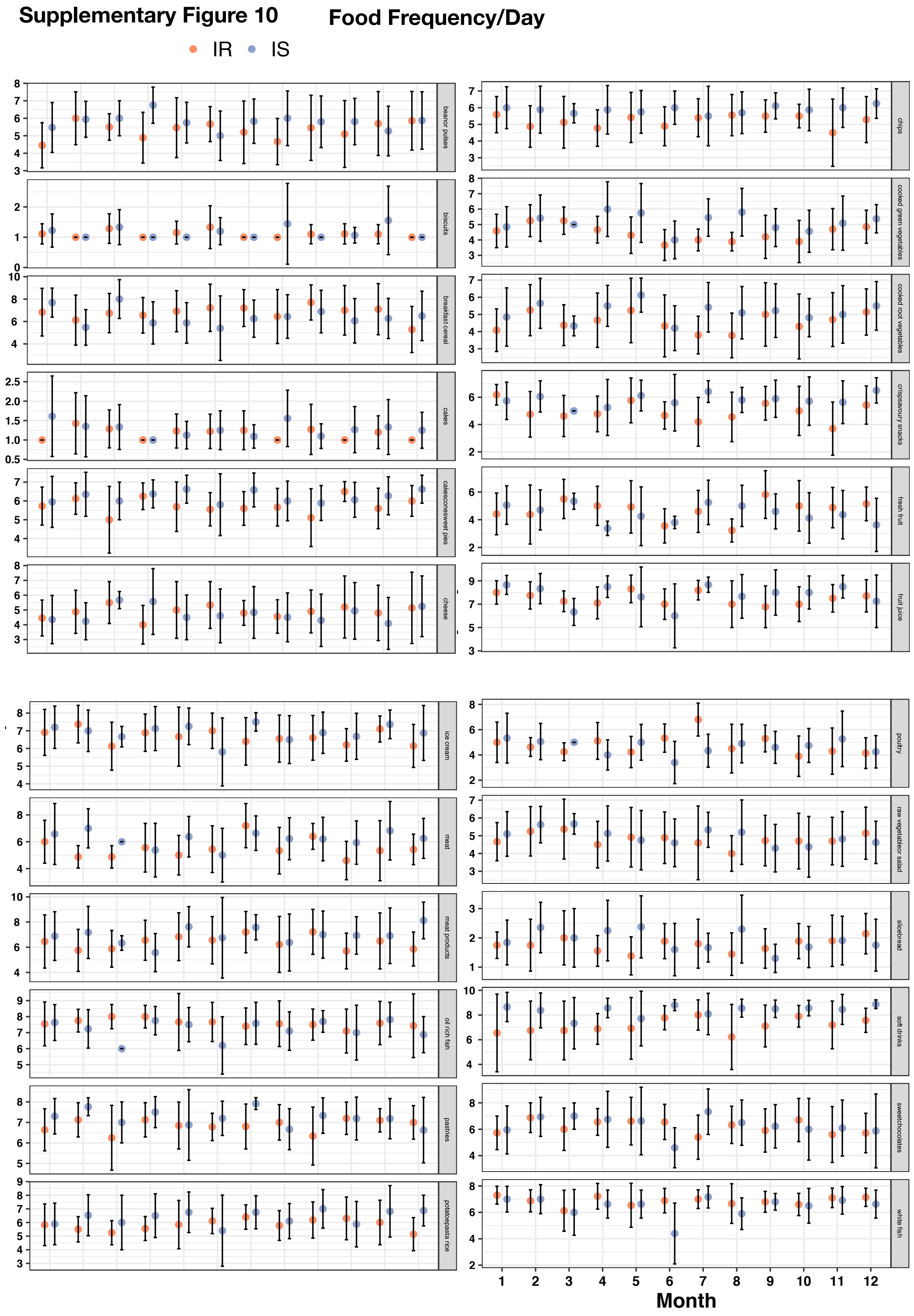

Supplementary Figure 10. Food categories frequencies between IR and IS groups throughout the year. The Y-axis shows the food frequency per day and X-axis show months of the year. Circle and whisker plots show median, and maximum and minimum values, respectively. Statistical significance was assessed by one-way ANOVA with random blocks. Blue circles represent IS group and orange circles represent IR group.

Supplementary Figure 11

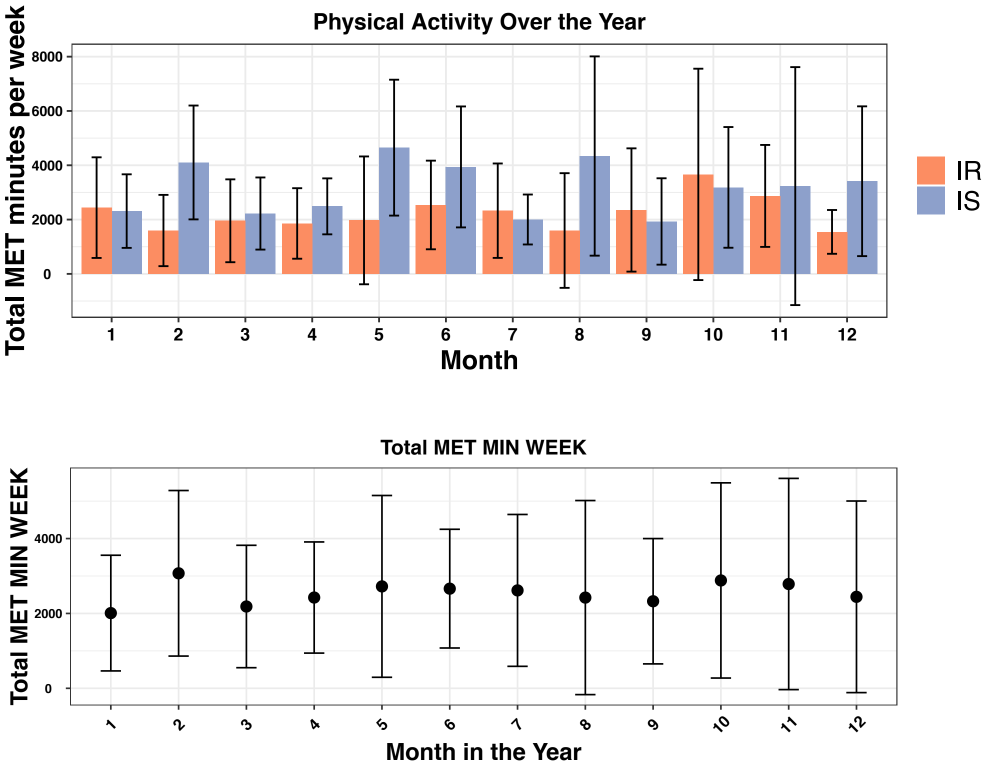

Supplementary Figure 11. Physical activities differences between IR and IS groups throughout the year. Y-axis show the total MET min per week and X-axis show the months of the year. Circle and whisker plots show median, and maximum and minimum values, respectively. Statistical significance was assessed by one-way ANOVA with random blocks.

Supplementary Figure 12

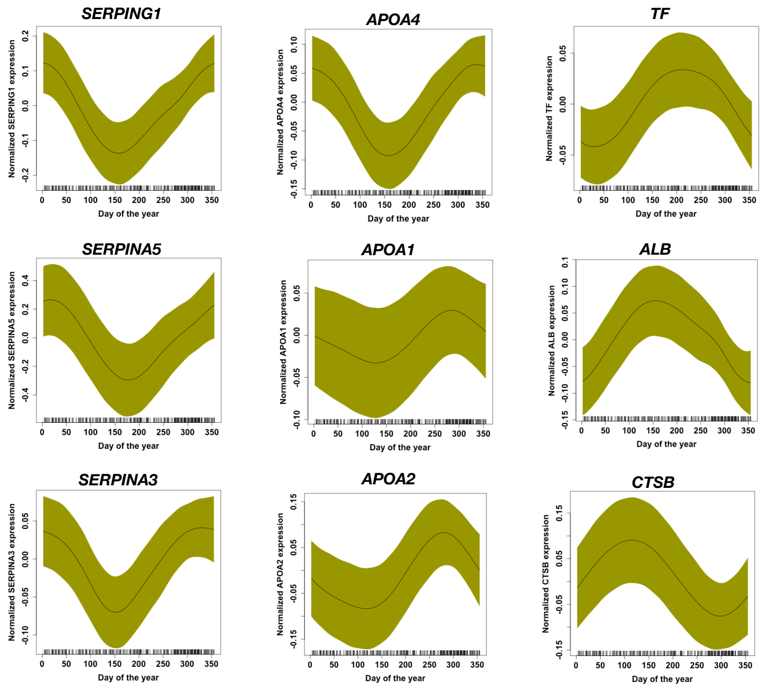

Supplementary Figure 12. Seasonal patterns of omics analytes known to be related with hibernations. The Y axis shows the days of the year and the X axis shows normalized expression level. The shaded area represents 95% confidence bounds computed as +-1.96 standard deviation of model coefficients. Standard deviations were derived from a maximum likelihood fit.

Supplementary Table 1: Description of the seasonal cohort characteristics

	# Subjects (105)
**Gender**	
**Male**	50
**Female**	55
**Ethnicity**	
**Caucasian**	70
**African American**	6
**Asian**	22
**Hispanic**	4
**Unknown**	3
**SSPG**	
**IR**	35
**IS**	31
**Untested**	39
**Age (years)**	54.5 (10)
**BMI (kg/m2)**	28.21 (4.368)

Continuous variable reported as range (avg ± sd) and categorical variable reported as N (%)

Supplementary Table 2: List of diseases with seasonality effects

Diseases	Pattern one (-log10(P))	Pattern two (-log10(P))
Hypertension	5.405888249	0
cardiovascular system	5.046593657	0
Metabolic acidosis	5.0405629	0
Disorder of blood pressure	5.021559967	0
Viral Infection	4.956226103	2.506058106
Schizophrenia spectrum disorder	4.549164733	0
Aggressive behavior	3.934206513	0
Seizures	3.919796264	0
Sleep pattern	3.894246404	0
Circadian rhythm	3.791116893	0
Atherosclerosis	3.694944905	0
Acne	0	3.14282868
Esterification of cholesterol	0	8.086453575
Inflammation of myocardium	0	3.560838325
Sjögren syndrome	0	3.947729745
Volume of urine	0	5.116111608

Supplementary Table 3: IR and IS cohort characteristics.

	IR (35)	IS (31)	*p*-value
**Gender**			0.57
**Male**	17 (48.5%)	12 (39%)	
**Female**	18 (51.5%)	19 (61%)	
**Ethnicity**			0.3497
**Caucasian**	23 (65%)	19 (61%)	
**African American**	1 (3%)	5 (16%)	
**Asian**	8 (23%)	6 (19%)	
**Hispanic**	2 (6%)	1 (4%)	
**Unknown**	1 (3%)	0 (0%)	
**Age (years)**	44 - 69 (56.74 ± 6.9)	36 - 67 (54.73 ± 8.44)	0.33
**BMI (kg/m^2)**	23.3-38.9 (30.1 ± 3.6)	21.4 - 33.3 (27.4 ± 3.4)	0.004
**SSPG**	151 - 276 (202 ± 36.4)	40 - 148 (89.52 ± 28.9)	3.30E-12

Continuous variable reported as range (avg ± sd) and categorical variable reported as N (%)

Supplementary Table 4: List of Total Pollen Counts - GAMM coefficients and P-values

Pollen Type	P-values	T1	T2	T3	T4	T5	T6	T7	T8
**Trees Pollens**	5.45E-18	25.73	34.81	21.2	4.62	-2.3	-2.434	-20.3	-35
**Grass Pollens**	2.95E-12	-3.42	-2.3	8.65	6.4	-0.7	-2.11	-3.36	-5
**Weed Pollens**	2.16E-09	-1.91	-2.76	1.16	3.67	3.59	1.944	-1.15	-2.8
**Mold Spores**	0.00060497	0.612	14.17	20.6	19.8	13.1	-0.143	-9.78	-14

Supplementary Table 5: Food consumption frequencies differences between IR and IS individuals.

NO.	Food type	p-value
1	slice bread	0.159822968
2	biscuits	0.466940517
3	cakes	0.340641827
4	breakfast_cereal	0.107785308
5	fresh_fruit	0.002487949
6	cooked_green_vegetables	0.075840475
7	cooked_root_vegetables	0.697135271
8	raw_vegetableor_salad	0.882331841
9	chips	0.497372163
10	potatoepasta_rice	0.570810148
11	meat	0.897163917
12	meat_products	0.854573044
13	poultry	0.292757724
14	white_fish	0.116964274
15	oil_rich_fish	0.450172205
16	cheese	0.372012863
17	beanor_pulses	0.743361482
18	sweetchocolates	0.551016756
19	ice_cream	0.63838028
20	crispsavoury_snacks	0.024429473
21	fruit_juice	0.048805601
22	soft_drinks	0.040375122
23	cakesconesweet_pies	0.059110423
24	pastries	0.071165653
