# Supplementary material for: Deep longitudinal multiomics profiling reveals two biological seasonal patterns in California
Source: Nat Commun. 2020 Oct 1;11:4933. doi: 10.1038/s41467-020-18758-1 (PMC7529769; doi:10.1038/s41467-020-18758-1)
Supplement: Supplementary file 3 — Reporting Summary [file 41467_2020_18758_MOESM3_ESM.pdf]

nature research

Corresponding author(s): Prof. Michael Snyder

Last updated by author(s): 2020/06/01

Reporting Summary

Nature Research wishes to improve the reproducibility of the work that we publish. This form provides structure for consistency and transparency in reporting. For further information on Nature Research policies, see Authors & Referees and the Editorial Policy Checklist .

Statistics

For all statistical analyses, confirm that the following items are present in the figure legend, table legend, main text, or Methods section.

n/a	Confirmed	
☐	☒	The exact sample size (n) for each experimental group/condition, given as a discrete number and unit of measurement
☐	☒	A statement on whether measurements were taken from distinct samples or whether the same sample was measured repeatedly
☐	☒	The statistical test(s) used AND whether they are one- or two-sided<br>*Only common tests should be described solely by name; describe more complex techniques in the Methods section.*
☐	☒	A description of all covariates tested
☐	☒	A description of any assumptions or corrections, such as tests of normality and adjustment for multiple comparisons
☐	☒	A full description of the statistical parameters including central tendency (e.g. means) or other basic estimates (e.g. regression coefficient) AND variation (e.g. standard deviation) or associated estimates of uncertainty (e.g. confidence intervals)
☐	☒	For null hypothesis testing, the test statistic (e.g. F, t, r) with confidence intervals, effect sizes, degrees of freedom and P value noted<br>*Give P values as exact values whenever suitable.*
☒	☐	For Bayesian analysis, information on the choice of priors and Markov chain Monte Carlo settings
☒	☐	For hierarchical and complex designs, identification of the appropriate level for tests and full reporting of outcomes
☒	☐	Estimates of effect sizes (e.g. Cohen's d, Pearson's r), indicating how they were calculated

Our web collection on statistics for biologists contains articles on many of the points above.

Software and code

Policy information about availability of computer code

Data collection	R 3.4 (specified in online methods for which analysis which version was used); TopHat 2.0.11, HTseq 0.6.1, DESEQ2 v3.5.
Data analysis	generalized additive mixed model (GAMM) in mgcv package, ggplot2 package , OmicsLonDA , Mfuzz package in R, ingenuity pathway analysis (IPA®, QIAGEN Inc.) , TopHat 2.0.11, HTseq 0.6.1, DESEQ2 v3.5

For manuscripts utilizing custom algorithms or software that are central to the research but not yet described in published literature, software must be made available to editors/reviewers. We strongly encourage code deposition in a community repository (e.g. GitHub). See the Nature Research guidelines for submitting code & software for further information.

Data

Policy information about availability of data

All manuscripts must include a data availability statement. This statement should provide the following information, where applicable:

- Accession codes, unique identifiers, or web links for publicly available datasets
- A list of figures that have associated raw data
- A description of any restrictions on data availability

Raw data included in this study are hosted on the NIH Human Microbiome 2 project site (https://portal.hmpdacc.org/) under the study T2D. The processed data of host and microbiome data used in this study are also available in supporting information file.

Field-specific reporting

Please select the one below that is the best fit for your research. If you are not sure, read the appropriate sections before making your selection.

- [x] Life sciences
- [ ] Behavioural & social sciences
- [ ] Ecological, evolutionary & environmental sciences

For a reference copy of the document with all sections, see nature.com/documents/nr-reporting-summary-flat.pdf

Life sciences study design

All studies must disclose on these points even when the disclosure is negative.

Sample size	Sample size for the main HMP project was calculated based on the pilot iPOP study findings of levels of autocorrelation in multiomics data and the number of timepoints needed per participant to reconstruct the time series with < 1% uncertainty. For this study, we used all iPOP participants with available clinical data.
Data exclusions	All available data was used for analyses. There was no data excluded from analyses.
Replication	This was an observational study which did not involve experiments.
Randomization	Participants were not randomized in this study. There was no allocation to groups, since there is no group-based analysis in the study. In our analyses, we controlled for BMI at time of consent and sex since these covariates were considered potential confounders.
Blinding	There was no blinding in this study because it is not relevant to the study (There is no allocation to groups or interventions in the study). Omics data was processed without knowledge of participants' clinical status.

Reporting for specific materials, systems and methods

We require information from authors about some types of materials, experimental systems and methods used in many studies. Here, indicate whether each material, system or method listed is relevant to your study. If you are not sure if a list item applies to your research, read the appropriate section before selecting a response.

Materials & experimental systems

n/a	Involved in the study	
[x]	[ ]	Antibodies
[x]	[ ]	Eukaryotic cell lines
[x]	[ ]	Palaeontology
[x]	[ ]	Animals and other organisms
[ ]	[x]	Human research participants
[x]	[ ]	Clinical data

Methods

n/a	Involved in the study	
[x]	[ ]	ChIP-seq
[x]	[ ]	Flow cytometry
[x]	[ ]	MRI-based neuroimaging

Human research participants

Policy information about studies involving human research participants

Population characteristics	This cohort includes 105 individuals (55 women and 50 men) with ages ranging from 25 to 75 years old. This is generally a healthy cohort and well characterized for glucose dysregulation using annual oral glucose tolerance tests (OGTTs), insulin resistance tests using a steady state glucose measurements (SSPG), Fasting glucose and hemoglobin A1c.
Recruitment	Participants were recruited via placement of advertisements in local newspapers and radio stations seeking "prediabetic volunteers" at risk for Type 2 diabetes for longitudinal multi-omic study. Screening in the CTRU entailed history and physical, anthropometric measurements, and fasting blood tests for exclusions including presence of anemia defined as hematocrit < 30, renal disease defined as creatinine > 1.5, history of any cardiovascular, malignancy, chronic inflammatory, psychiatric disease, and history of any bariatric surgery or liposuction.
Ethics oversight	Stanford University Institutional Review Board (IRB 23602).

Note that full information on the approval of the study protocol must also be provided in the manuscript.
