# Supplementary material for: Deep longitudinal multiomics profiling reveals two biological seasonal patterns in California
Source: Nat Commun. 2020 Oct 1;11:4933. doi: 10.1038/s41467-020-18758-1 (PMC7529769; doi:10.1038/s41467-020-18758-1)
Supplement: Supplementary file 4 — Description of Additional Supplementary Files [file 41467_2020_18758_MOESM4_ESM.pdf]

Description of Additional Supplementary Files

Supplementary Code 1: The code for seasonality modeling (*GAMM* model)

Supplementary Code 2: The code for seasonal clustering

Supplementary Data 1: Cohort residential locations in California.

Supplementary Data 2: List of omics analytes with seasonality effects.

Supplementary Data 3: List of omics analytes in pattern one and two.

Supplementary Data 4: List of canonical pathways with seasonality effects.

Supplementary Data 5: List of molecules profiled by luminex assay.

Supplementary Data 6: List of clinical lab tests.

Supplementary Data 7: List of omics analytes with their GAMM coefficients.

Supplementary Data 8: List of meteorological data measurements.

Supplementary Data 9: Correlation of seasonal patterns with microbiome, clinical lab tests and meteorological measurements.

Supplementary Data 10. List of airborne fungi counts and their GAMM coefficients and P-values**.**

Supplementary Data 11. Physical activities differences between IR and IS individuals throughout the year.

Supplementary Data 12: Significant time intervals of differentially abundant/expressed omics features between IR/IS

Supplementary Data 13: Total airborne pollen counts data

Supplementary Data 14: Total airborne fungi counts data

Supplementary Data 15: Dietary habits data

Supplementary Data 16: The International Physical Activity Questionnaire **(**IPAQ) Data
