# Supplementary material for: Deep longitudinal multiomics profiling reveals two biological seasonal patterns in California
Source: Nat Commun. 2020 Oct 1;11:4933. doi: 10.1038/s41467-020-18758-1 (PMC7529769; doi:10.1038/s41467-020-18758-1)
Supplement: Supplementary file 2 — Peer Review File [file 41467_2020_18758_MOESM2_ESM.pdf]

Reviewers' comments:

Reviewer #1 (Remarks to the Author):

1. Why was insulin sensitivity/insulin resistance chosen as a specific disease for analysis? There are many factors known to influence the balance between IR and IS that were not measured here (e.g., exercise level, daily caloric intake, dietary diversity, etc., each of which can vary with season), making it difficult to interpret the seasonal changes reported.

2 Do any of the seasonal omic variants have counterparts among hibernators? There are well-known changes in specific (plasma) proteins among hibernators that alter hemostasis, metabolism, thermoregulation, etc. These evolutionary adaptations may, at least in part, explain some of the variation reported here and should be explored.

3. How generalizable are the seasonal influences determined in the Bay Area? Given the more dramatic meteorological changes in season in other parts of the world—with, in some cases, truly four seasons—it is difficult to assess the usefulness of these data beyond the 37th (N) parallel.

4. How do seasonal changes in daylight influence the changes observed and what links the circadian changes mentioned to the seasonal changes?

5. On page 11, the description of Sjogren syndrome is incomplete, and emphasizes less frequent elements than typically observed.

6. How does seasonal variation in pollen count affect the nasal microbiome? Can pollen sequences be detected in the nasal samples?

7. On page 13, the relationship of increased platelet counts to insulin resistance is not widely accepted.

Reviewer #2 (Remarks to the Author):

In this paper, in order to understand the molecular and microbial changes that occur during seasonal variations, Snyder and colleagues profiled 105 participants in the San Francisco bay area over four years, in a longitudinal and multi-omics study. From the study, the authors identified more than 1000 seasonal variations in omics analytes and clinical measures. The different molecules grouped into two major seasonal patterns which correlate with peaks in late spring and late fall/early winter in the San Francisco Bay Area. Additionally, the authors identified molecules and microbes that demonstrated 33 different seasonal patterns in insulin sensitive and insulin resistant individuals.

This is a very interesting study. The study design is novel, addressed an important question. The data produced are extensive and very valuable. The impact of the study is perceived to be very high. I have the following comments:

• It is mentioned that samples were drawn over up to four years. but the data shown in all results only cover one year of time. Why not show the trend over the entire course of the study? That way, the seasonal trend will become more apparent.

• Demographic information such as age, gender, ethnicity has been collected. I wonder whether the authors have studied these factors to see if any of these factors has any impact on the seasonal patterns. I understand the sample size may be limited hence low power. But it would be interesting to

see if any gene, metabolite, or other molecular and
microbial markers show any gender-specific effects or pattern.

• As pointed out by the authors, human activity levels also differ by season. So the overall seasonal patterns may be resulted from weather-related factors such as temperature, humidity, or from non-weather-related factors such as outdoor activity time and diet. I wonder whether there is anyway that such differences can be detected from the multi-omics data.

• I wonder whether the authors are able to detect other period pattern in their data that might not be seasonal. For example, menstrual period in women.

• The author says that the optimal number of clusters is 2. But it seems that Figure 2a. indicates any number >2 gives pretty much the same result. May be Supplementary Fig 2b is a better plot to use.

minor:
• It would be nice if the authors can make all their analysis codes public available.

Reviewer #3 (Remarks to the Author):

The authors describe a multi-omic approach to definition of seasonal parameters in a longitudinal study of 105 healthy volunteers in California.

In general, this study could have the potential to add valuable insight into human seasonality, and in particular cross-talk involving multi-omic approaches. By way of background, there is a very large and voluminous literature on seasonal parameters in humans, much of it not cited here for reasons of possible space limitation. However, there are a number of concerns as the magnitude and precision of the data.

Specifically:
1) The studies have employed a Generalized Additive Mixed Model (GAMM), which in plain language creates statistics from different non-overlapping parameters. The model is based on the use of a smoothing term (cyclic cubic regression spline; It is not clear in the methods what the difference is between GAM and GAMM). One difficulty with this approach is that it difficult to see how such model-fitting complies to raw data. For instance, in Figures 1c, 2c, 3d etc, plotting of the raw un-manipulated data on the same axis would be invaluable in adding confidence to the reader. It is also not clear what the shaded areas represent – confidence limits? If so, how where these calculated – specifically against variance in the raw data set of as a parameter derived from the spline-fit model? Are the data on microbiomes used in the clustering analysis – hard to see from the paper.

2) It is very hard to see what is model and what is real. In Fig 1, is this conceptual or based on real data? If real – it looks there are 3 clusters. Fig 1b could be placed in Supplementary as methological.

3) What was the basis for selection of parameters shown in Fig 1c?

4) In general, the figures are hard to follow from text to figure and not presented in order of appearance. In some cases, it is not clear whether the figure presents new data, or a pathway drawn from the literature without citation ie 2g. If the former in this case, what is the magnitude and direction of effect? Or are all of these parameters that individually change by season? If so, how do we ascribe confidence to these data? It is striking how marked the apparent differences appear to be – ie in Fig 3a, there appear to be no scores for some parameters in patterns 1 vs 2 –but in the real world

these markers are always expressed. So, what smoothing parameters to set such high stringency?

5) In marked contrast to photoperiodic animal species where there are specific neuroendocrine circuits and known pathways/cell types involved and their role defined, in humans the situation is much less clear. It was none-the-less striking that not one reference to the much better defined literature on animal models was cited, and perhaps reference to one major review would help.

6) Critically, animal studies clearly show that there are profound time-of-day and day-night cycles that influence the overall seasonal response – see literature on melatonin, earlier concepts on the specific role of the circadian clock as an interval timer, links to thyroid hormone signaling (driving metabolic processes) and immune links as well. The over-riding message from these studies is that correct anchoring of sample time to the prevailing photophase is crucial. Just one example will suffice. Data on Per1 are shown (Fig 1c). It is most probable that the seasonal rhythm here is an artifact, based on differences in time intervals from dawn to sampling times. To take this to other data sets shown, how is controlled for in the study. It is rather extraordinary that no reference is made to the prevailing light-dark cycle, or possible effect of seasonal changes in phase angle on the magnitude of the effects observed. Fig 3 makes reference to "seasonal parameters" but not photoperiod or sampling data time of day. If the latter data were not collected and the study not controlled for this effect, then much of what is presented is inevitably open-ended. This is for me a real issue in this paper, as absence of a correction for photophase time makes interpretation hard.

In conclusion, the study is potentially interesting. But it is virtually impossible to link the smoothing parameters to the real data. Further, the data may not be anchored in the real world unless underlying diurnal phase-angle components can be convincingly addressed. At very least this latter issue needs to be convincingly addressed in discussion and not dismissed within a single sentence and no reference.

Nature Communications Reviewer's comments:

We would like to thank the reviewers and the respected editor for all the critics and guidance's, which help strengthen the manuscript substantially. Please find below our responses.

Reviewers' comments:

Reviewer #1 (Remarks to the Author):

1. Why was insulin sensitivity/insulin resistance chosen as a specific disease for analysis? There are many factors known to influence the balance between IR and IS that were not measured here (e.g., exercise level, daily caloric intake, dietary diversity, etc., each of which can vary with season), making it difficult to interpret the seasonal changes reported.

We thank the reviewer for raising this comment. Our cohort is generally healthy. However, since our cohort was well characterized with the gold standard test for peripheral insulin resistance, the modified Insulin Suppression Test (IST) (Pei et al. 1994; PMID: 7988789) via measuring the steady-state plasma glucose (SSPG), it made it possible to categorize participants as insulin sensitive (IS) and insulin resistance (IR). IR is an important risk factor for many metabolic diseases such as T2D and CVD. We analyzed how seasonal changes occur between Insulin Sensitive and Insulin Resistance individuals. We are aware that there are many factors known to influence the balance between IR and IS. In our analysis, we accounted for BMI, as higher BMI is strongly associated with IR (Wilcoxon signed-rank test p-value = 0.006328). We also accounted for the age and health status of each individual. In our updated manuscript we added data about dietary habits, which were assessed every three months using the Dietary Targets Monitor survey. The survey asks about the frequency of consumption of a variety of foods including fish, meat, cheese, fruits and vegetables, starchy foods as well as sweets, and savory snacks. In total we have 25 different food categories (see the Method section page 27 Lines 782-791 and Supporting Data file 2). None of the food categories were significantly different between IR and IS groups throughout the year (One-way ANOVA with random blocks, P-value >0.05, Supplemental Table S15, Supplemental Figure S10) . In our analysis we used Subject ID as a random effect to account for different numbers of samples per subject. On the other hand, physical activity measured in total metabolic equivalent of task (MET) minutes expended in the past week, is significantly different

between IR and IS, specifically in February, May, June, and August (One-way ANOVA with random blocks, P-value = 0.01787, Supplemental Figure S11). However, a post-hoc analysis of all omics features that were identified by OmicsLonDA to be significantly different between IR and IS showed that they are not associated with physical activity.

2 Do any of the seasonal omic variants have counterparts among hibernators? There are well-known changes in specific (plasma) proteins among hibernators that alter hemostasis, metabolism, thermoregulation, etc. These evolutionary adaptations may, at least in part, explain some of the variation reported here and should be explored.

We thank the reviewer for raising this point. We looked into this and found several genes and proteins in our seasonal analytes that have been known to be linked with hibernation periods. We have now included a section in the discussion part addressing this point. Please see page 15 lines 423-429 and Supp. Figure S12.

3. How generalizable are the seasonal influences determined in the Bay Area? Given the more dramatic meteorological changes in season in other parts of the world—with, in some cases, truly four seasons—it is difficult to assess the usefulness of these data beyond the 37th (N) parallel.

We thank the reviewer for bringing up this concern. Our data shows extensive seasonal variations across different omes in respect to seasons. It is striking to see that even in the Bay area, known for having mild seasons, there are many analytes that display seasonal changes. This study highlights the importance of conducting similar studies in other areas with dramatic seasonal changes and we believe represents a novel approach and type of study. By holistically capturing different types of omics data as well as nasal and gut microbiota over four years as well as integrating meteorological data and airborne pollens and fungi from the same area we can developed a snapshot of what types of seasonal patterns exist and affect human biochemical and physiological functions.

As mentioned by the reviewer, we expect that regions with four distinct seasons will experience a dramatic change in temperature and possibly show even broader seasonal variations. Our study has important health implications, for example in interpreting clinical lab health data or evaluating the impact of different airborne pollens on allergies. We have a note in the discussion section to mention this issue (Please see page 16, Lines 449-451).

4. How do seasonal changes in daylight influence the changes observed and what links the circadian changes mentioned to the seasonal changes?

This is a great point. The samples were collected at approximately the same time for each person and all were collected between 7am – 10am over the course of four years. Thus, the variation in sample collection time is low. As we collected one sample per visit from fasted individuals, we can only analyze seasonal changes and not the solar diurnal cycles.
It is of note that due to conducting many tests on every sample, we collected a large volume of blood in one drawing from fasted individuals, making it hard to ask patients to have a second visit on the same day. We have added a note in the discussion section to mention this concern. Please see page 16, Lines 443-449).

5. On page 11, the description of Sjogren syndrome is incomplete, and emphasizes less frequent elements than typically observed.

We have more details about Sjogren's syndrome reflecting the common characteristics. Please see page 13 line 346-347.

6. How does seasonal variation in pollen count affect the nasal microbiome? Can pollen sequences be detected in the nasal samples?

Thank you for this insightful comment. As our nasal microbiome profiling was based on 16S sequencing, we were not able to detect pollen and fungi from our sequencing data. However, we have now included pollen counts from tree pollens, weed pollens, mold spores, grass pollens, as well as airborne fungi from the same region as our participants live and incorporated these into our analyses (San Francisco Bay area). Specifically, we have analyzed the seasonal patterns of airborne pollens and fungi and have correlated their patterns with our omics seasonal patterns. (Please see Figure 5; Page 8 and 14; Supplemental Figure S9, Supplemental Tables S13 and S14). The new data has strengthened our results especially in regard to our previously reported observations related to allergies. It also provides information in regard to the onset, duration and severity of the airborne pollen and fungi season in the Bay area and it’s relationship with omics seasonal patterns.

Assuming the reviewer is asking about mold counts, unfortunately, we won't be able to detect any fungi in our nasal microbiome profiling. We used 16S rRNA sequencing to profile the nasal microbiome, which is limited to bacterial taxa.

7. On page 13, the relationship of increased platelet counts to insulin resistance is not widely accepted.

We thank the reviewer for bringing this concern to our attention. We have a note in the discussion section to reflect this concern. We also removed the previous sentence that discussed this relationship. Please see page 15 Line 420-421.

Reviewer #2 (Remarks to the Author):

In this paper, in order to understand the molecular and microbial changes that occur during seasonal variations, Snyder and colleagues profiled 105 participants in the San Francisco bay area over four years, in a longitudinal and multi-omics study. From the study, the authors identified more than 1000 seasonal variations in omics analytes and clinical measures. The different molecules grouped into two major seasonal patterns which correlate with peaks in late spring and late fall/early winter in the San Francisco Bay Area. Additionally, the authors identified molecules and microbes that demonstrated 33 different seasonal patterns in insulin sensitive and insulin resistant individuals.

This is a very interesting study. The study design is novel, addressing an important question. The data produced are extensive and very valuable. The impact of the study is perceived to be very high.

We thank the reviewer for the positive comment.

I have the following comments:

• It is mentioned that samples were drawn over up to four years. but the data shown in all results only cover one year of time. Why not show the trend over the entire course of the study? That way, the seasonal trend will become more apparent.

The samples are drawn over up to four years. However, in order to strengthen the seasonal analysis, the four years period has been aggregated and mapped to one-year long timeframe (Figure 1b). It will increase the power of the GAMM model in detecting seasonal variations. We have added this information to the figure legend for figure 1 for more clarification.

• Demographic information such as age, gender, ethnicity has been collected. I wonder whether the authors have studied these factors to see if any of these factors has any impact on the seasonal patterns. I understand the sample size may be limited hence low power. But it would be interesting to see if any gene, metabolite, or other molecular and microbial markers show any gender-specific effects or pattern.

We thank the reviewer for this comment. We have analyzed as much demographic information as we could collect. Our analysis indicated that the BMI and health status (IR or IS) were significant. We accounted for BMI and health status in our analysis as covariates. Please refer our response to the first reviewer for all details of demographics/diet/exercise association with omics features in IR/IS analysis.
Please note that gender was not a significant contributor based on Wilcoxon signed-rank test.

• As pointed out by the authors, human activity levels also differ by season. So the overall seasonal patterns may be resulted from weather-related factors such as temperature, humidity, or from non-weather-related factors such as outdoor activity time and diet. I wonder whether there is any way that such differences can be detected from the multi-omics data.

We thank the reviewer for bringing up this concern. As the respected reviewer mentioned, seasons are a complex phenomenon. Seasons by definition are “a period of the year characterized by particular conditions of weather, temperature, ecology and the amount of daylight”. In our study we tried to find omics (the host and microbiome) seasonal changes associated with the weather-related factors that define seasons. However, we analyzed the non-weather-related conditions and found out that the health status (IR/IS) and BMI have a significant contribution and therefore we have accounted for these factors in our analysis. We have also included diet data and physical activity data collected from participants and have accounted for them in our analysis of IR and IS individuals. Please refer our response to the first reviewer for all details of demographics/diet/exercise association with omics features in IR/IS analysis.

• I wonder whether the authors are able to detect other period patterns in their data that might not be seasonal. For example, the menstrual period in women.

We thank the reviewer for raising this insightful point. It would have been very interesting to find changes related to other patterns (e.g. menstrual period in women).

However, this information has not been collected. We will add a note in the discussion part to point to other cyclic events like menstrual period in women for future study designs. Please see page 16 lines 447-449 for the added note.

• The author says that the optimal number of clusters is 2. But it seems that Figure 2a. indicates any number >2 gives pretty much the same result. Maybe Supplementary Fig 2b is a better plot to use.

We have replaced Figure 2a with Figure S2b.

minor:
• It would be nice if the authors can make all their analysis codes public available.

We provide the analysis code as supporting information file attached to this manuscript and on GitHub.

Reviewer #3 (Remarks to the Author):

The authors describe a multi-omic approach to the definition of seasonal parameters in a longitudinal study of 105 healthy volunteers in California.

In general, this study could have the potential to add valuable insight into human seasonality, and in particular crosstalk involving multi-omic approaches. By way of background, there is a very large and voluminous literature on seasonal parameters in humans, much of it not cited here for reasons of possible space limitation. However, there are a number of concerns as to the magnitude and precision of the data.

We thank the reviewer for the positive comment. Due to limitation in space we kept the introduction short. We have now included additional sentences with new references. Please see page 3 Lines 47-57.

Specifically:
1) The studies have employed a Generalized Additive Mixed Model (GAMM), which in plain language creates statistics from different non-overlapping parameters. The model

is based on the use of a smoothing term (cyclic cubic regression spline; It is not clear in the methods what the difference is between GAM and GAMM). One difficulty with this approach is that it is difficult to see how such model-fitting complies to raw data. For instance, in Figures 1c, 2c, 3d etc, plotting of the raw un-manipulated data on the same axis would be invaluable in adding confidence to the reader. It is also not clear what the shaded areas represent – confidence limits? If so, how were these calculated – specifically against variance in the raw data set as a parameter derived from the spline-fit model? Are the data on microbiomes used in the clustering analysis – hard to see from the paper.

(A) We thank the reviewer for mentioning this concern. We used the `gamm` function in the `mgcv` package that allows fitting smoothing terms (a cyclic cubic splines) to model seasonal time-series data. As you mentioned, GAMM stands for Generalized Additive Mixed Model, which is different from GAM (Generalized Additive Model). We used GAMM in our analysis to account for personal variations by modeling Subject as random effect. The returned fitted model from the `gamm` function has two objects: gam and lme. The gam object is the one that contains all information about the smoothing parameters. To remove such confusion in the updated manuscript, we referred to the fitted model as GAMM.

(B) We have also mapped the residuals on the smoothing spline for several features as an example below. Panel A shows an example of an analyte with significant seasonal component (p-value =1.27E-08) based on our data. This feature is also known to have seasonality according to the previous research studies (Higgins, T. et al. 2009). Panel B shows an example of an analyte that does not carry a significant seasonal component (p-value=0.72) and is not known to have seasonality component based on previous researches.

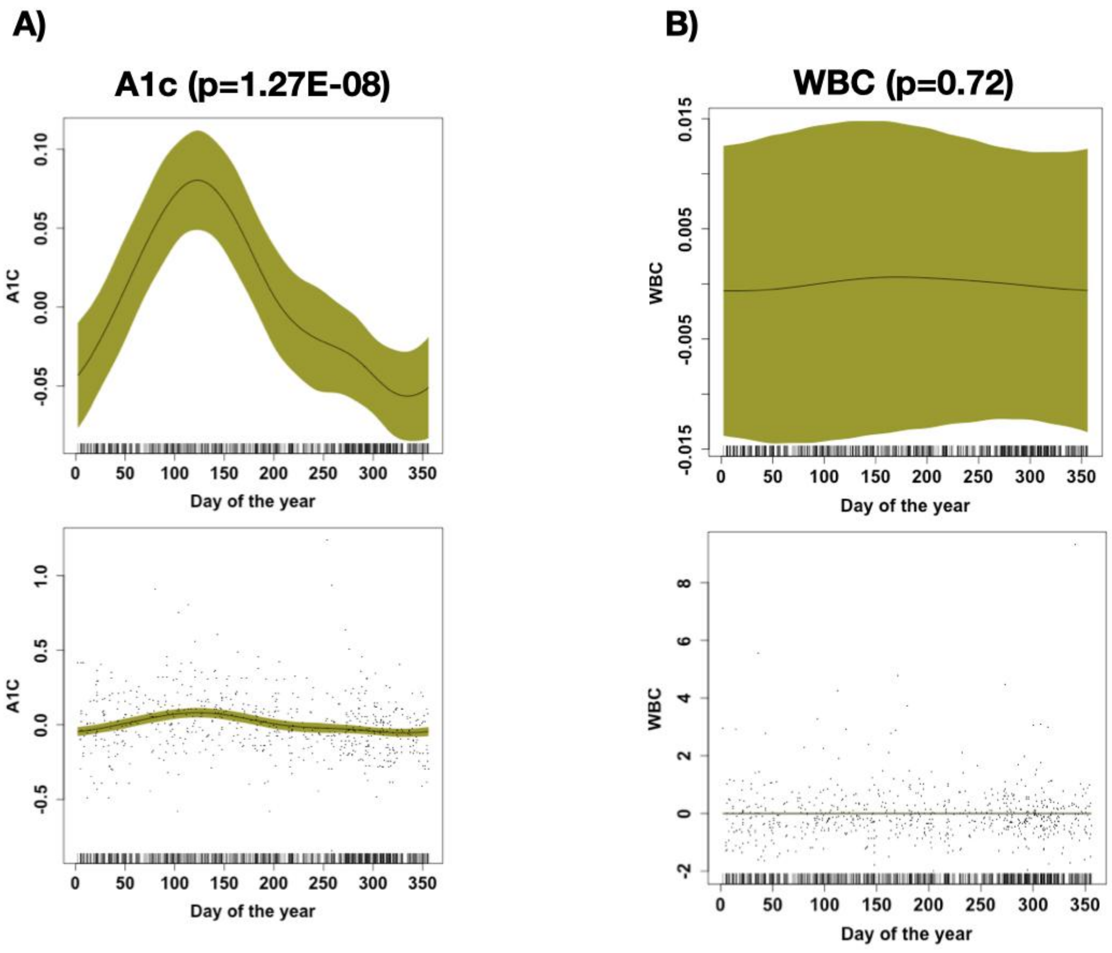

In the main document we showed the upper panel, as it gives a better resolution for the seasonality component.

(C) We appreciate the reviewer for pointing out the lack of this information describing our plots in Figure 1c and other figures. As the reviewer expected, it's computed as a parameter derived from the spline-fit model. We updated the caption in all relevant figures to explain our procedure for deriving standard deviations and confidence bounds. It reads as “The shaded area represents 95% confidence bounds computed as +-1.96 standard deviation of model coefficients. Standard deviations were derived from a maximum likelihood fit”.

(D) Yes, Microbiome data are included in the plot.

2) It is very hard to see what is a model and what is real. In Fig 1, is this conceptual or based on real data? If real – it looks there are 3 clusters. Fig 1b could be placed in Supplementary as methological.

Figure 1a is a schematic representation of our approach. We have removed the inner picture in Fig1a to avoid confusion. We have also noted the figure's legend to make this clear.
We think Figure 1b is helpful to show a summary of our cohort and its characteristics, therefore it may help the readers to see it in the main figure.

3) What was the basis for selection of parameters shown in Fig 1c?

The selection is based on p-value. Figure 1c shows examples of molecules selected from different omes, which show significant seasonal effects based on GAMM model ($p<0.05$). It shows examples of molecules known to exhibit seasonal changes based on literature (e.g. A1c, RDW, HDL, LDL/HDL,PER1,genus-Firmicutes) as well as new ones (e.g. SIGLEC15, IP10, IL1RAP,C2,C5,IL5) discovered through our study.

4) In general, the figures are hard to follow from text to figure and not presented in order of appearance. In some cases, it is not clear whether the figure presents new data, or a pathway drawn from the literature without citation ie 2g. If the former in this case, what is the magnitude and direction of effect? Or are all of these parameters that individually change by season? If so, how do we ascribe confidence to these data? It is striking how marked the apparent differences appear to be – ie in Fig 3a, there appear to be no scores for some parameters in patterns 1 vs 2 –but in the real world these markers are always expressed. So, what smoothing parameters to set such high stringency?

We thank the reviewer for this concern. A) We have now made sure that figures follow the text.
B). All the figures come from our data, except the schematic figure in Fig1a.
Fig 2g shows examples of pathways based on omics analytes that we identified in our study. These omics analytes carry significant seasonal components ($p<0.05$). We applied ingenuity pathway analysis (IPA) to search for enriched pathways in our list of omics analytes (input file) with significant seasonality components. The IPA enrichment algorithm uses an enrichment score based on Fisher's exact test P value. The P value represents the significance of the overlap between observed (the input file) and predicted regulated molecules in those pathways. Pathways that show significant enrichment ($p<0.05$) are shown in our manuscript in Fig2e and 2f. In Fig2g we have shown omics analytes related to some of these pathways that show a significant enrichment ($p<0.05$).

5) In marked contrast to photoperiodic animal species where there are specific neuroendocrine circuits and known pathways/cell types involved and their role defined, in humans the situation is much less clear. It was none-the-less striking that not one reference to the much better defined literature on animal models was cited, and perhaps reference to one major review would help.

We thank the reviewer for pointing to this concern. Due to the limitations in space we made our introduction section short. We have now mentioned more references in the introduction section including the photoperiodic aspects. Please see page 3 lines 47-57.

6) Critically, animal studies clearly show that there are profound time-of-day and day-night cycles that influence the overall seasonal response – see literature on melatonin, earlier concepts on the specific role of the circadian clock as an interval timer, links to thyroid hormone signaling (driving metabolic processes) and immune links as well. The over-riding message from these studies is that correct anchoring of sample time to the prevailing photophase is crucial. Just one example will suffice. Data on Per1 are shown (Fig 1c). It is most probable that the seasonal rhythm here is an artifact, based on differences in time intervals from dawn to sampling times. To take this to other data sets shown, how is controlled for in the study. It is rather extraordinary that no reference is made to the prevailing light-dark cycle, or possible effect of seasonal changes in phase angle on the magnitude of the effects observed. Fig 3 makes reference to "seasonal parameters" but not photoperiod or sampling data time of day. If the latter data were not collected and the study not controlled for this effect, then much of what is presented is inevitably open-ended. This is for me a real issue in this paper, as absence of a correction for photophase time makes interpretation hard.

We thank the reviewer for this comment. We acknowledge that the dark-light cycle may have an influence on our findings in this study and we have noted this issue in the discussion part. However, we tried to minimize this effect by taking the samples from the same individual at the same time throughout the study. In addition, all the samples have been taken between 7am-10am at the Stanford Clinical and Translational Research Unit (CTRU) from fasted individuals. We have a note in the discussion section (page 16, Lines 443-449) to reflect this concern. We also noted in the discussion section to better control for photoperiodism in future studies.

In conclusion, the study is potentially interesting. But it is virtually impossible to link the smoothing parameters to the real data. Further, the data may not be anchored in the real world unless underlying diurnal phase-angle components can be convincingly

addressed. At very least this latter issue needs to be convincingly addressed in discussion and not dismissed within a single sentence and no reference.

We thank the reviewer for this comment. As mentioned above, we acknowledge that the light-dark cycle may have influenced our findings and have a note in the discussion part. However, we would like to bring the respected reviewer's attention to these points:
First: As we collected one sample per individual per visit, we can only analyze seasonal changes and not the daily circadian rhythm changes or diurnal cycles.
It is of note that due to conducting many tests on every sample, we collected a large volume of blood in one drawing from fasted individuals, making it hard to ask patients to have a second visit on the same day (dark hours). However, in order to minimize the effect of daylight cycles on our seasonal changes' assessment, for each person the samples were collected at approximately the same time and all were collected between 7am - 11am over the course of four years. Thus, the variation in sample collection time is low. However, we acknowledge that a better control setting for photoperiodism is needed for future studies and we mentioned this in the text page 16.

Second: We have selected analytes that have a seasonal component (measured by smoothing parameters) based on p-values ($p<0.05$). We agree with the reviewer that seasons are a complex phenomenon and there are multiple factors that may contribute to our observations in this study, including daylight cycle and air temperature, to name a few. In our study, we have done our best to consider all the possible factors that may have a contribution. We have also included a note in the discussion part to address this concern and recommendations for future studies.

REVIEWERS' COMMENTS:

Reviewer #1 (Remarks to the Author):

None.

Reviewer #2 (Remarks to the Author):

All of my previous comments have been addressed sufficiently.

Reviewer #3 (Remarks to the Author):

The authors have provided an improved MS which clarifies earlier points raised. I have one remaining point, and apologise if I did not make this sufficiently clear in the initial report
In the discussion the authors state:

"It is of note 446 that our data does not capture changes associated with solar diurnal cycle and dark-light cycle as we collected one sample per each visit from fasted participants. However, we tried to minimize the effect of solar diurnal cycle on our seasonal changes assessment by taking samples at the same time for each fasted participant (7am - 10am) for every visit over the course of four years."

My point is that by collecting at the same solar time of day (ZT) over the year, one is collecting at different ZTs relative to dawn and dusk. We know already that seasonal changes in lighting have significant effects on the phase of human physiology, not the least sleep, and there is a mass of data linking this to a wide range of other physiological parameters (see for instance papers by Roenneberg and colleagues). The phase of sampling relative to the time of light-onset and offset will likely impact on some of the parameters. So, perhaps the authors might consider the additional sentence at the bottom of above:
"Since sampling took place at the same time of day, but at different times relative to the onset of the light dark cycle, and since the latter is known to impact on the phase of both sleep and human physiology (take your pick from multiple reviews on the topic), the seasonal differences we report may be accounted for in part by sampling at different phases of the seasonal light cycle."

<u>Nature Communications Reviewer's comments:</u>

We would like to thank the reviewers and the editor for all the critics and guidance's, which help strengthen the manuscript substantially over the course of review process. Please find below our responses.

REVIEWERS' COMMENTS:

Reviewer #1 (Remarks to the Author):

None.

We thank the reviewer again for his/her previous comments that helped to strengthen our manuscript.

Reviewer #2 (Remarks to the Author):

All of my previous comments have been addressed sufficiently.

We thank the reviewer again for his/her previous comments that helped to strengthen our manuscript.

Reviewer #3 (Remarks to the Author):

The authors have provided an improved MS which clarifies earlier points raised. I have one remaining point, and apologise if I did not make this sufficiently clear in the initial report
In the discussion the authors state:

"It is of note that our data does not capture changes associated with solar diurnal cycle and dark-light cycle as we collected one sample per each visit from fasted participants. However, we tried to minimize the effect of solar diurnal cycle on our seasonal changes assessment by taking samples at the same time for each fasted participant (7am - 10am) for every visit over the course of four years."

My point is that by collecting at the same solar time of day (ZT) over the year, one is collecting at different ZTs relative to dawn and dusk. We know already that seasonal changes in lighting have significant effects on the phase of human physiology, not the least sleep, and there is a mass of data linking this to a wide range of other physiological parameters (see for instance papers by Roenneberg and colleagues). The phase of sampling relative to the time of light-onset and offset will likely impact on some of the parameters. So, perhaps the authors might consider the additional sentence at the bottom of above:

"Since sampling took place at the same time of day, but at different times relative to the onset of the light dark cycle, and since the latter is known to impact on the phase of both sleep and human physiology (take your pick from multiple reviews on the topic), the seasonal differences we report may be accounted for in part by sampling at different phases of the seasonal light cycle."

We thank the reviewer for this clarification and his/her previous comments that helped to strengthen our manuscript. We added the suggested sentence in our discussion part. Please see page 15: lines 429-431.
